# Sex differences in mitochondrial gene expression during viral myocarditis

**DOI:** 10.1186/s13293-024-00678-0

**Published:** 2024-12-18

**Authors:** Damian N. Di Florio, Gabriel J. Weigel, David J. Gorelov, Elizabeth J. McCabe, Danielle J. Beetler, Katie A. Shapiro, Katelyn A. Bruno, Isha Chekuri, Angita Jain, Emily R. Whelan, Gary R. Salomon, Sami Khatib, Natalie E. Bonvie-Hill, Jessica J. Fliess, Presley G. Giresi, Charwan Hamilton, Cameron J. Hartmoyer, Varsini Balamurugan, Ashley A. Darakjian, Brandy H. Edenfield, S. Christian Kocsis, Christopher J. McLeod, Leslie T. Cooper, Étienne Audet-Walsh, Michael J. Coronado, Jon Sin, DeLisa Fairweather

**Affiliations:** 1 Department of Cardiovascular Medicine, Mayo Clinic, Jacksonville, FL USA; 2 Center for Clinical and Translational Science, Mayo Clinic, Rochester, MN USA; 3 Mayo Clinic Graduate School of Biomedical Sciences, Mayo Clinic, Jacksonville, FL USA; 4 Division of Cardiovascular Medicine, Department of Medicine, University of Florida, Gainesville, FL USA; 5 Department of Cancer Biology, Mayo Clinic Jacksonville, Jacksonville, FL USA; 6 Endocrinology - Nephrology Research Division, CHU de Québec - Université Laval Research Center, Québec, QC Canada; 7 Cytokinetics Inc, South San Francisco, CA 94080 USA; 8 Department of Biological Sciences, University of Alabama, Tuscaloosa, AL USA; 9 Department of Immunology, Mayo Clinic, Jacksonville, FL USA; 10 Department of Medicine, Mayo Clinic, Jacksonville, FL USA

**Keywords:** Inflammation, Innate immunity, Coxsackievirus B3, Estrogen-related receptor alpha/ERRα, Mitochondria, Electron transport chain, ChIP

## Abstract

**Background:**

Myocarditis is an inflammation of the heart muscle most often caused by viral infections. Sex differences in the immune response during myocarditis have been well described but upstream mechanisms in the heart that might influence sex differences in disease are not completely understood.

**Methods:**

Male and female BALB/c wild type mice received an intraperitoneal injection of heart-passaged coxsackievirus B3 (CVB3) or vehicle control. Bulk-tissue RNA-sequencing was conducted to better understand sex differences in CVB3 myocarditis. We performed enrichment analysis and functional validation to understand sex differences in the transcriptional landscape of myocarditis and identify factors that might drive sex differences in myocarditis.

**Results:**

As expected, the hearts of male and female mice with myocarditis were significantly enriched for pathways related to an innate and adaptive immune response compared to uninfected controls. Unique to this study, we found that males were enriched for inflammatory pathways and gene changes that suggested worse mitochondrial electron transport function while females were enriched for pathways related to mitochondrial homeostasis. Mitochondria isolated from the heart of males were confirmed to have worse mitochondrial respiration than females during myocarditis. Unbiased TRANSFAC analysis identified estrogen-related receptor alpha (ERRα) as a transcription factor that may mediate sex differences in mitochondrial function during myocarditis. Transcript and protein levels of ERRα were confirmed as elevated in females with myocarditis compared to males. Differential binding analysis from chromatin immunoprecipitation (ChIP) sequencing confirmed that ERRα bound highly to select predicted respiratory chain genes in females more than males during myocarditis.

**Conclusions:**

Females with viral myocarditis regulate mitochondrial homeostasis by upregulating master regulators of mitochondrial transcription including ERRα.

**Supplementary Information:**

The online version contains supplementary material available at 10.1186/s13293-024-00678-0.

## Abstract (plain language summary)

Many viruses can infect the heart. Immune cells go to the heart to get rid of the virus but sometimes this response can damage the heart. When immune cells go to the heart it is called “myocarditis,” which means ‘myo’ for muscle and ‘carditis’ for heart inflammation. More men get myocarditis after viral infections than women. When we study viral infection in mice as a model of human disease, we see the same sex differences as in humans. To know why this happens, we gave mice a viral infection and looked at how their hearts changed. We found that a special gene acts like a *light switch* to turn on the genes that help cell energy factories called “mitochondria.” Females used this light switch to turn on the genes that help mitochondria, but males did not and turned most of these genes off. We learned that the light switch works better in females compared to males, so it may play an important role in protecting female’s hearts during myocarditis. More research is needed to better understand how this light switch works. Understanding how to turn on mitochondria genes in the heart could help doctors also do this in men after a viral infection to prevent myocarditis and save patients’ lives.

**Supplementary Information:**

The online version contains supplementary material available at 10.1186/s13293-024-00678-0.

## Highlights

• Females maintain mitochondrial homeostasis with less severe myocarditis.
• Several master regulators of mitochondrial homeostasis are elevated in females.
• Transcription factor ERRα RNA and protein are elevated in female hearts.
• ERRα binds mitochondrial genes more in females during myocarditis.

**Supplementary Information:**

The online version contains supplementary material available at 10.1186/s13293-024-00678-0.

## Background

Myocarditis is an inflammation of the myocardium, or muscle tissue of the heart, and a leading cause of sudden cardiac death in persons under 50 years of age [1, 2]. The Global Burden of Disease (GBD) study from 2019 reported 1.8 million cases of myocarditis world-wide [2]. A 2014 Swedish study reported myocarditis at an incidence of 8.6 people per 100,000 [3]. Several epidemiological studies estimated at least a 15-fold increased incidence of myocarditis/ perimyocarditis from severe acute respiratory syndrome coronavirus-2 (SARS-CoV-2) infection during the coronavirus disease 2019 (COVID-19) pandemic [4, 5]. Prior to the pandemic, coxsackievirus B3 (CVB3) was the leading suspected cause of myocarditis in the United States. Myocarditis can progress to dilated cardiomyopathy (DCM) in susceptible individuals and in mouse models of viral and autoimmune myocarditis [6–8]. Animal models have demonstrated that upregulated profibrotic remodeling genes during acute myocarditis (day 10 post infection/pi) lead to the development of fibrosis and ventricular dilation/ DCM from day 35 pi onwards [9, 10]. Chronic heart failure during DCM leads to heart transplants in a significant proportion of patients [11, 12]. A lack of disease-specific therapies aside from heart failure medications provides impetus for identification of novel biomarkers and therapeutic targets with the goal of earlier detection and more targeted treatment.

The incidence and severity of myocarditis is greater in cis-males (referred to hereafter as males) than cis-females (referred to hereafter as females) in humans and mouse models [8]. The GBD study reported a mortality rate in patients with myocarditis aged 35–39 of 4.4 per 100,000 in women and 6.1 per 100,000 in men, indicating that more men die of myocarditis than women worldwide [2]. We previously reported a sex ratio of 3.5 males to 1 female among patients with biopsy confirmed myocarditis [1]. Men are more likely to develop cardiac fibrosis and progress to DCM after myocarditis compared to women [10, 11, 13]. We, and others, previously reported that testosterone promotes a proinflammatory and profibrotic response in an autoimmune model of CVB3 myocarditis while estrogen is cardioprotective [1, 10, 14, 15]. The goal of this study was to better understand sex differences in CVB3 myocarditis using bulk-tissue RNA sequencing (RNAseq). We found major sex differences in transcriptional programming related to cardiac mitochondrial homeostasis during myocarditis and identified estrogen-related receptor alpha (ERRα) as a transcription factor that may be responsible for mediating the sex difference.

## Results

### Myocardial inflammation is increased in males compared to females

**Fig. 1:**
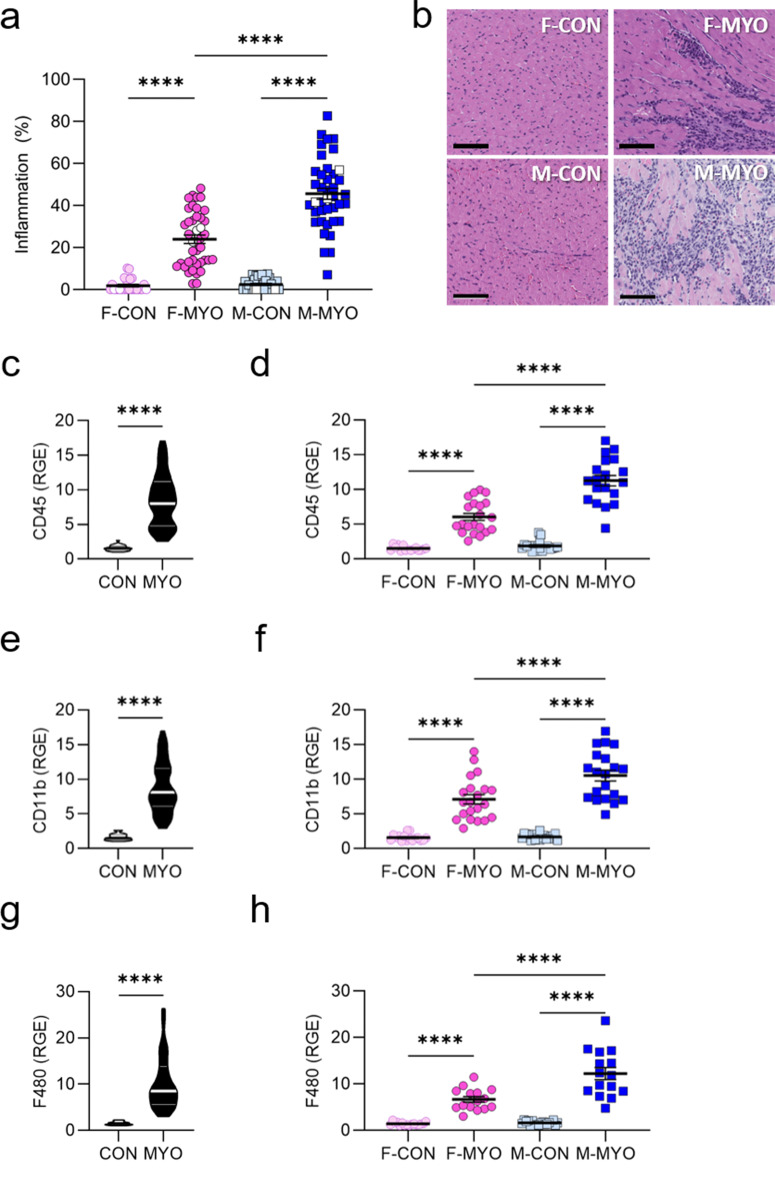
Myocardial inflammation is increased in males compared to females. **a**, Myocarditis severity (% inflammation) between female controls (F-CON, *n* = 27), females with myocarditis (F-MYO, *n* = 41), male controls (M-CON, *n* = 30), and males with myocarditis (M-MYO, *n* = 40); white symbols indicate the samples chosen for RNA sequencing (*n* = 3); **b**, representative heart sections (scale bars = 80 μm); **c**-**h**, relative gene expression (RGE) for F-CON (*n =* 15–16), F-MYO (*n =* 15–21), M-CON (*n =* 18), and M-MYO (*n =* 15–20)

We first examined inflammation in males versus females and uninfected PBS vehicle controls to confirm sex differences and to select samples for RNA sequencing. As we have shown previously [16], males in our autoimmune CVB3 model develop significantly more inflammation than females (*p* = 0.0001) according to histological assessment whereas vehicle controls did not develop myocarditis (Fig. 1a). Representative examples of histology for each group are shown in Fig. 1b. We confirmed major immune cell types in the heart of males vs. females with myocarditis compared to controls using qRT-PCR. We found total immune cells (CD45, *p* < 0.0001), complement receptor 3 activated myeloid cells (CD11b, *p* < 0.0001), and macrophages (F4/80, *p* < 0.0001) were increased in males with myocarditis compared to females with myocarditis (Fig. 1c-h), as expected [16]. Thus, males have greater cardiac inflammation during autoimmune CVB3 myocarditis than females.

### Females upregulate while males downregulate gene pathways related to mitochondrial homeostasis during myocarditis

**Fig. 2:**
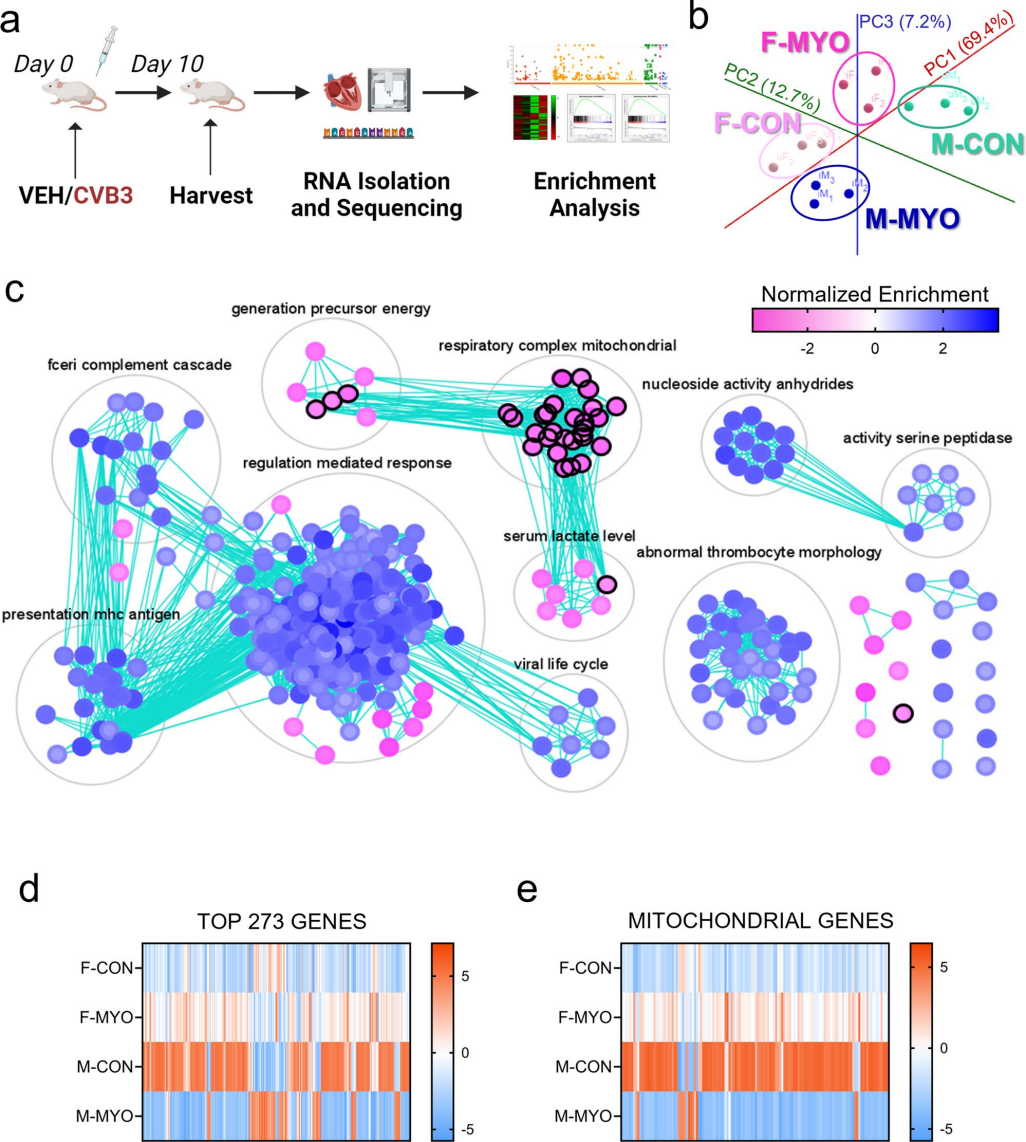
RNA sequencing reveals sex differences in immune and mitochondrial genes. **a**, RNA-sequencing experimental pipeline; **b**, principal component analysis plot showing female controls (F-CON), females with myocarditis (F-MYO), male controls (M-CON), and males with myocarditis (M-MYO); **c**, results from GSEA pre-ranked plotted on Cytoscape with Enrichment Map and AutoAnnotate, pink = F-MYO and blue = M-MYO, nodes circled in black are mitochondrial-related pathways; heat map for **d**, the top 273 most differentially expressed genes and **e**, mitochondrial genes between all four groups

We then examined sex differences in myocarditis using bulk-tissue RNAseq (an overview of the experimental design is illustrated in Fig. 2a). Samples chosen for RNAseq are depicted with white symbols in Fig. 1a. PCA analysis revealed good separation between groups and high similarity within groups (Fig. 2b). To better understand the mechanisms underlying sex differences in myocarditis, we performed gene set enrichment analysis (GSEA) of RNAseq data comparing males and females with myocarditis versus controls. When we compared females and males with myocarditis, we found males with myocarditis (blue) had significantly enriched clusters (i.e., auto-annotated grouped gene sets) for the following gene pathways compared to females: regulation mediated (immune) response, viral life cycle, presentation MHC antigen, Fc-receptor complement cascade, nucleoside activity anhydrides, abnormal thrombocyte morphology, and activity of serine peptidases (Fig. 2c). Females with myocarditis (pink) had significantly enriched clusters for the following gene pathways compared to males: respiratory complex mitochondrial, generation precursor energy, and serum lactate levels (Fig. 2c). Non-super-clustered gene sets (i.e., nodes) and their identities are displayed in Additional File 1: Figure S1. These data indicate that females with myocarditis have higher expression of mitochondrial respiratory transcripts than males. In contrast, males have higher expression of immune system genes compared to females, which is consistent with histology findings (Fig. 1).

We used Cytoscape to generate heat maps from RNA sequencing data for the top 273 differentially expressed genes between males and females with myocarditis or controls (Fig. 2d). These data revealed distinct gene profiles between each group with 200 of the top 273 genes being increased in females during myocarditis compared to males with myocarditis (Fig. 2d). Females with myocarditis upregulated 216 genes compared to female controls in contrast to males with myocarditis that downregulated 210 genes compared to male controls (Fig. 2d).

To better understand sex differences in genes associated with mitochondrial function, we selected mitochondrial gene sets in Cytoscape to generate a heat map containing 132 differentially expressed and mitochondrial-specific genes comparing females to males with myocarditis or controls (Fig. 2e). The mitochondrial gene expression differences between controls and by sex were very similar to the findings of the top 273 genes. Females with myocarditis had higher expression of 118 of 132 mitochondrial genes compared to males with myocarditis (Fig. 2e). Females upregulated 119 of 132 mitochondrial genes during myocarditis compared to female controls while males downregulated 120 mitochondrial genes compared to male controls (Fig. 2e). These data demonstrate that males with myocarditis have decreased mitochondrial-related transcriptional support whereas females with myocarditis have increased mitochondrial homeostasis during myocarditis.

**Fig. 3:**
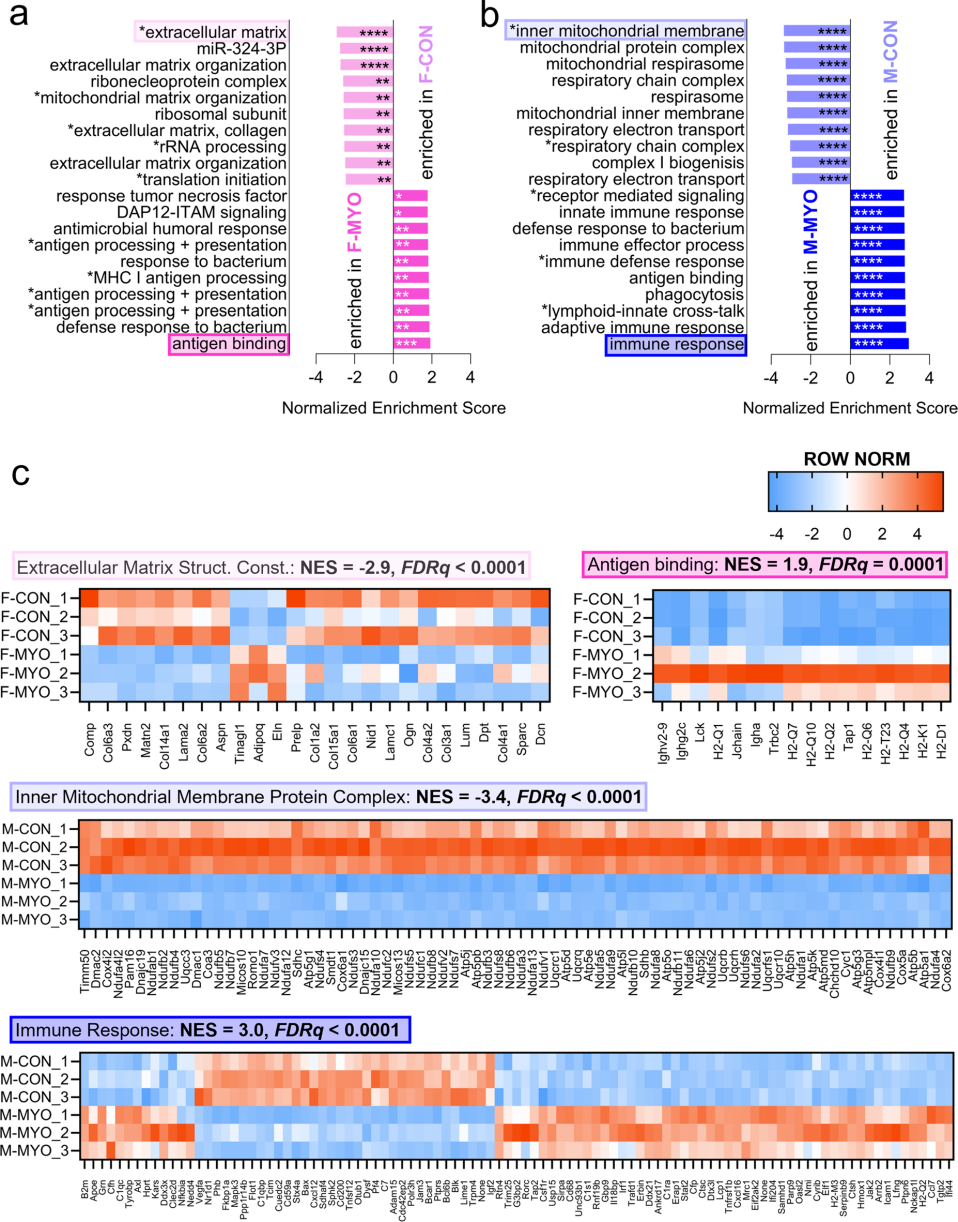
Sex-specific gene expression pathways during myocarditis. The top ten most enriched pathways from GSEA ranked by normalized enrichment score comparing **a**, female controls (F-CON) to females with myocarditis (F-MYO), **b**, male controls (M-CON) to males with myocarditis (M-MYO) (pathway text marked with astricts indicate abbreviated pathway names, see supplement for abbreviations and full pathway names) **c**, Row normalized heatmaps for the most enriched pathways from F-CON vs. F-MYO and M-CON vs. M-MYO, respective to colors highlighting pathways in (**a**) and (**b**). NES = normalized enrichment score, Extracellular Matrix Structural (Struct.) Constituent (Const.). * *FDRq <* 0.05, ** *FDRq* < 0.01, *** *FDRq* < 0.001, **** *FDRq* < 0.0001

To better understand gene pathways that differed by sex during myocarditis we plotted the top ten significant gene pathways from GSEA ranked by normalized enrichment score (NES) for control versus myocarditis (Fig. 3a, b) and by sex (Fig. 3c). We found that uninfected female hearts were enriched for gene sets related to mitochondrial and cellular homeostasis (Fig. 3a). Increased expression of transcripts related to immune activation such as “antigen processing and interaction” were found in females during myocarditis (Fig. 3a). Males with myocarditis transitioned from mitochondrial homeostasis in uninfected hearts to a proinflammatory immune response during myocarditis (Fig. 3b). We generated heatmaps corresponding to highlighted top significantly enriched pathways in the female control versus female myocarditis comparison (extracellular matrix structural constituent and antigen binding, respectively) and for the male control versus male myocarditis comparison (inner mitochondrial membrane protein complex and immune response, respectively) which can be found with NES and false discovery rate (FDRq) values in Fig. 3c.

**Fig. 4:**
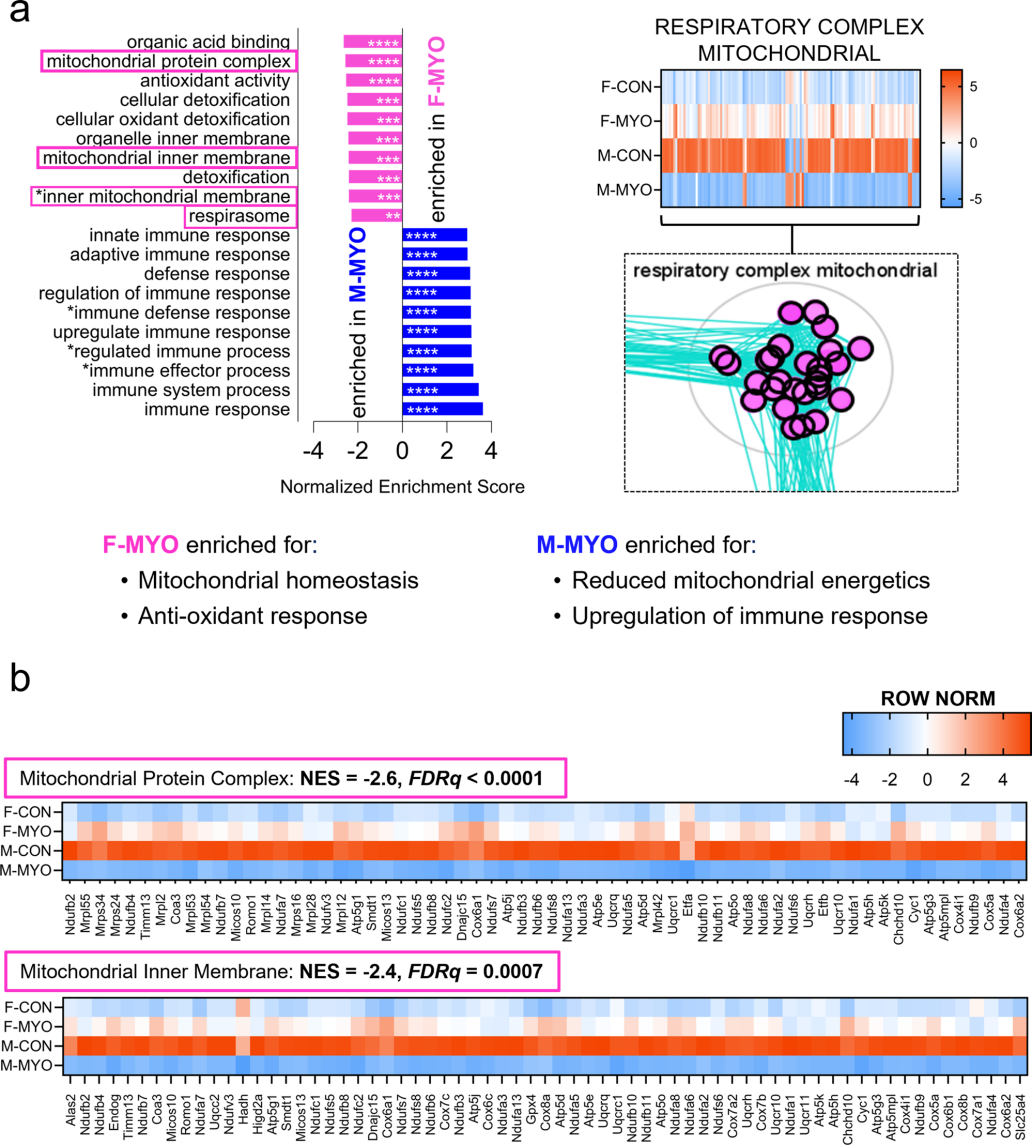
Sex differences in differential gene expression pathways during myocarditis. The top ten most enriched pathways from GSEA ranked by normalized enrichment score comparing **a**, females with myocarditis (F-MYO) and males with myocarditis (M-MYO) and heatmap of auto-annotated cluster of pathways/nodes describing the mitochondrial respiratory complex, **b**, Row normalized heatmaps for pathways highlighted in pink for F-MYO (from F-MYO vs. M-MYO comparison; other 2 pathways share common genes with those in (b) and are available in Additional File 1: Figure S3). * *FDRq <* 0.05, ** *FDRq* < 0.01, *** *FDRq* < 0.001, **** *FDRq* < 0.0001

A direct comparison of females to males with myocarditis revealed that females were enriched for pathways related to mitochondrial homeostasis and anti-oxidant responses while males were enriched for pathways related to the innate and adaptive immune responses (Fig. 4a). We generated a heatmap showing all four groups of genes and gene sets in the super-cluster auto annotated as “respiratory complex mitochondrial”, which contained enriched gene sets related to the mitochondrial respiratory chain. Similar to the findings of the heatmap of all mitochondrial genes in Fig. 2e, females had higher expression of 114 of 127 genes in this super-cluster (Fig. 4a). Females with myocarditis upregulated 115 genes compared to female controls and males with myocarditis downregulated 116 genes compared to male controls (Fig. 4a). We generated heatmaps and highlighted mitochondrial enriched gene sets, which were significantly more enriched in females with myocarditis compared to males with myocarditis including mitochondrial protein complex (NES = -2.6, *FDRq < 0.0001*) and mitochondrial inner membrane (NES = -2.4, *FDRq = 0.0007*) (Fig. 4b). Inner mitochondrial membrane protein complex (NES = -2.4, *FDRq = 0.0007*) and respirasome (NES = -2.3, *FDRq = 0.004*) were also significantly enriched in females with myocarditis and mostly contained common genes with the pathways shown in Fig. 4b; these heatmaps can be found in Additional File 1: Figure S2. Thus, aside from sex differences in immune changes during myocarditis, which have been well characterized in the past, the main difference in cardiac transcript enrichment between males and females with myocarditis occurred in pathways related to mitochondrial function.

**Fig. 5:**
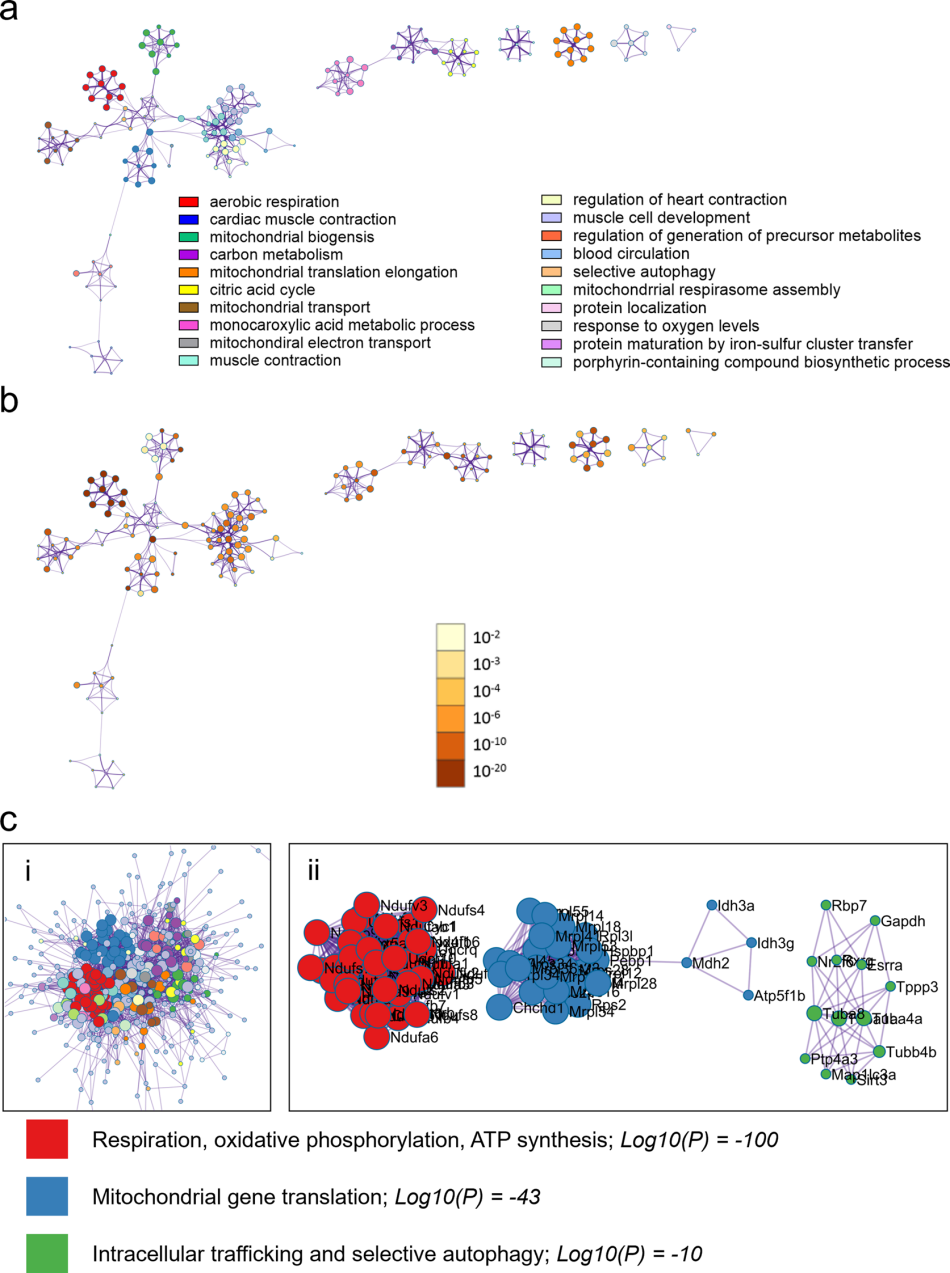
Females with myocarditis are enriched for pathways related to mitochondrial respiration compared to males with myocarditis. Metascape enrichment results for females with myocarditis (comparing females and males with myocarditis) **a**, the top enriched pathways colored by cluster/pathway and **b**, by p-value. Top enriched pathways **c**, Protein-protein interaction analysis clustered by interaction outside of pathways (i) and interaction inside pathways (ii). Log10(P) vals are derived by averaging the Log10(P) vals for the 3 MCODE annotations, rounded to whole number with colors indicating respective pathways. Images in (i) are cropped to show the bulk of pathways and interactors and the top 3 pathways only are shown in (ii)

**Fig. 6:**
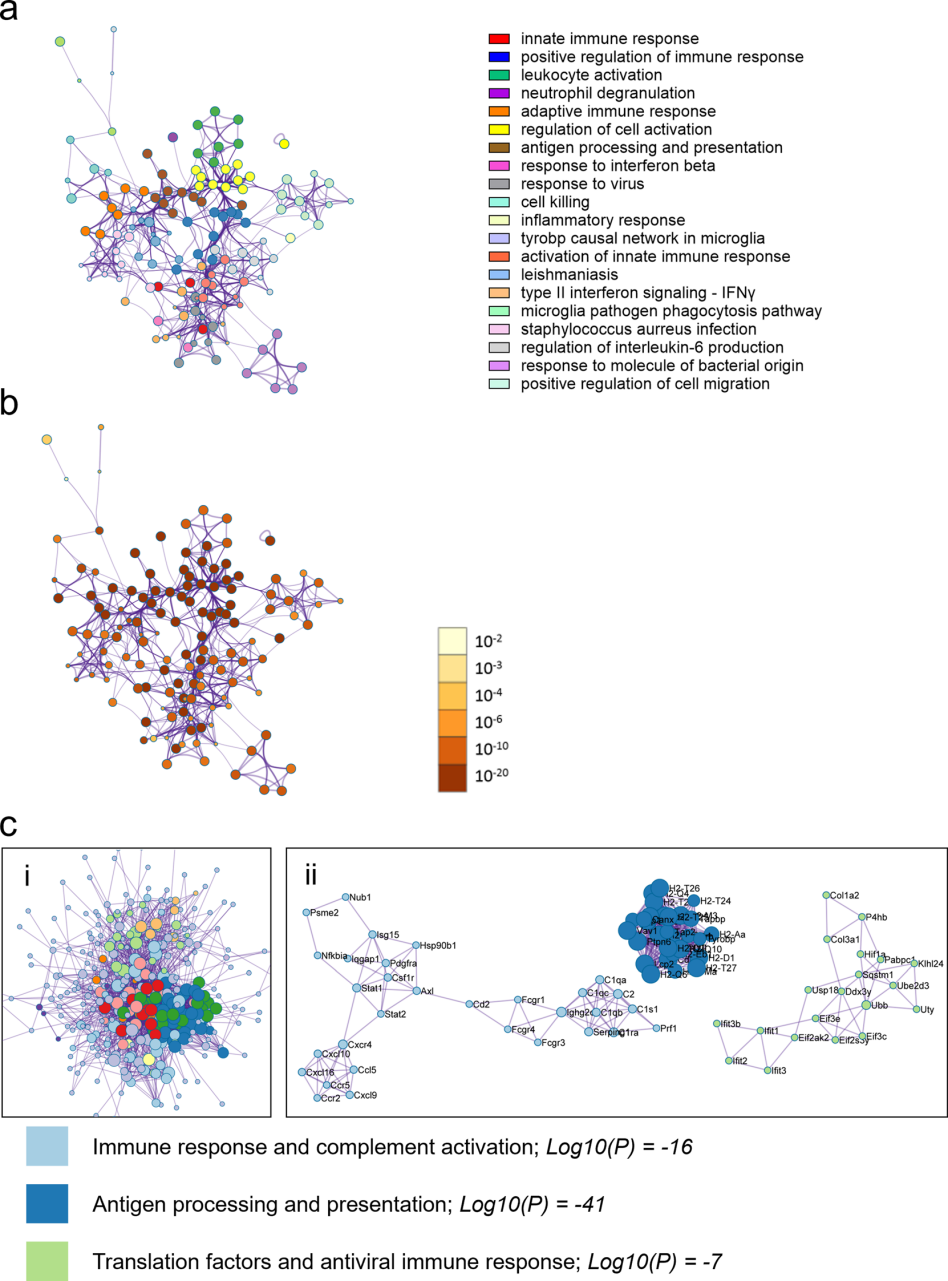
Males with myocarditis are enriched for pathways related immune activation compared to females with myocarditis. Metascape enrichment results for males with myocarditis (comparing females and males with myocarditis) **a**, the top enriched pathways colored by cluster/pathway and **b**, by p-value. Top enriched pathways **c**, Protein-protein interaction analysis clustered by interaction outside of pathways (i) and interaction inside pathways (ii). Log10(P) vals are derived by averaging the Log10(P) vals for the 3 MCODE annotations, rounded to whole number with colors indicating respective pathways. Images in (i) are cropped to show the bulk of pathways and interactors and the top 3 pathways only are shown in (ii)

To ensure that the observed sex differences in mitochondrial transcriptional enrichment were not a result of the enrichment method performed (i.e., GSEA Pre-Ranked), we additionally performed enrichment analysis using Metascape [17]. Metascape enrichment for females with myocarditis compared to controls versus males with myocarditis compared to controls were broadly similar to GSEA enrichment findings in Fig. 2 and can be found in Additional File 1: Figures S3-6. When we directly compared females with myocarditis to males with myocarditis, we confirmed that females with myocarditis were significantly enriched for pathways supporting mitochondria homeostasis and cell energetics (e.g., respiration, oxidative phosphorylation, ATP synthesis; Log10(P) = -100) (Fig. 5) and males with myocarditis were enriched for pathways related to upregulation of the immune response (Fig. 6). Importantly, MCODE_1 clustering of protein-protein interaction analysis by pathway, comprised of Reactome.org enrichment terms for respiratory electron transport (R-MMU-611105, and R-MMU-163200) and Gene Ontology oxidative phosphorylation (GO:0006119) pathways, were all enriched at Log10 p values of -100 further highlighting that mitochondrial pathways were the primary enrichment signature in females with myocarditis compared to males with myocarditis (Fig. 5c).

**Fig. 7:**
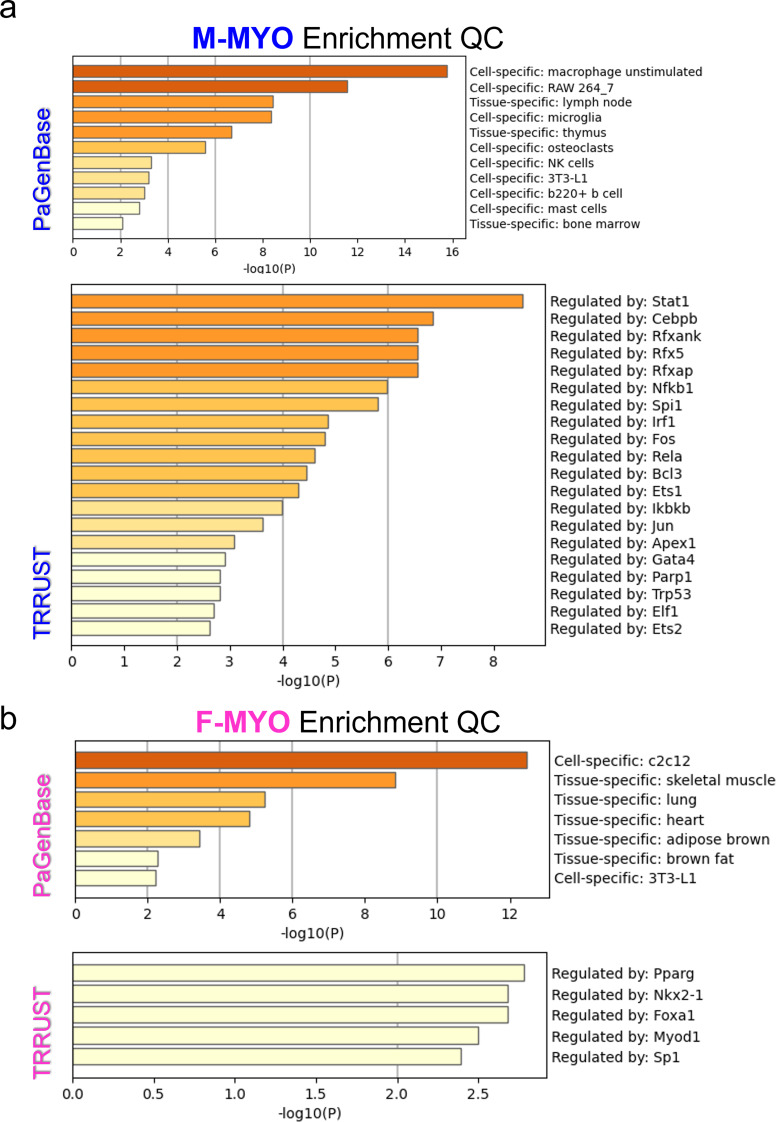
Metascape Enrichment QC shows cell specific signals and identifies potential transcriptional regulators of sex differences in myocarditis. **a**, Enrichment quality control (QC) for males with myocarditis (M-MYO) shows cell specific enrichment signals from Pattern Gene Database (PaGenBase) and suggested transcription factors from Transcriptional Regulatory Relationships Unraveled by Sentence-based Text mining (TRRUST) and for **b**, females with myocarditis (F-MYO)

Enrichment quality control metrics from Metascape revealed cell-specific signatures for males with myocarditis compared to females with myocarditis which mirrored the known prevalence of immune cells in the heart with highest to lowest being macrophages, T cells, natural killer (NK cells), B cells and mast cells (Fig. 7a) [16]. The most enriched transcription factor in males with myocarditis was signal transducer and activator of transcription (STAT)1, which is known to mount interferon (IFN) and T helper (Th)1/M1 immune responses that clear CVB3 infection during myocarditis [18, 19] (Fig. 7a). In contrast, females with myocarditis were more enriched for myoblasts (c2c12), myocytes and other cardiac cell components with an absence of immune cells compared to males with myocarditis (Fig. 7b). In contrast, the top enriched transcriptional regulator in females with myocarditis was peroxisome proliferator-activated receptor gamma (Pparg), a transcriptional regulator that promotes oxidative phosphorylation, antioxidant defense, mitochondrial biogenesis and regulates T cell responses [20, 21].

In Table S1, we display the top 5 most enriched gene sets from gProfiler comparing females and males with myocarditis, which were primarily comprised of gene sets related to mitochondrial homeostasis. The top enriched pathways from gProfiler for each source were: electron transfer activity (*p* = 2.68 E-37) (Gene Ontology (GO): Molecular Functions/MF), electron transport chain (*p* = 1.62 E-44) (GO: Biological Process/BP), mitochondrial inner membrane (*p* = 5.25 E-62) (GO: Cellular Component/CC), oxidative phosphorylation (*p* = 6.37 E-37) (Kyoto Encyclopedia of Genes and Genomes/ KEGG), and the citric acid cycle (*p* = 1.66 E-35) and respiratory electron transport/ ATP synthesis (*p* = 3.77 E-35) (Reactome/Reactome.org) (Additional File 1: Table S1). Overall, these results indicate that females maintain mitochondrial homeostasis during myocarditis while mitochondrial function in males plummets during myocarditis.

### Males with myocarditis have lower expression of electron transport chain genes compared to females

**Fig. 8:**
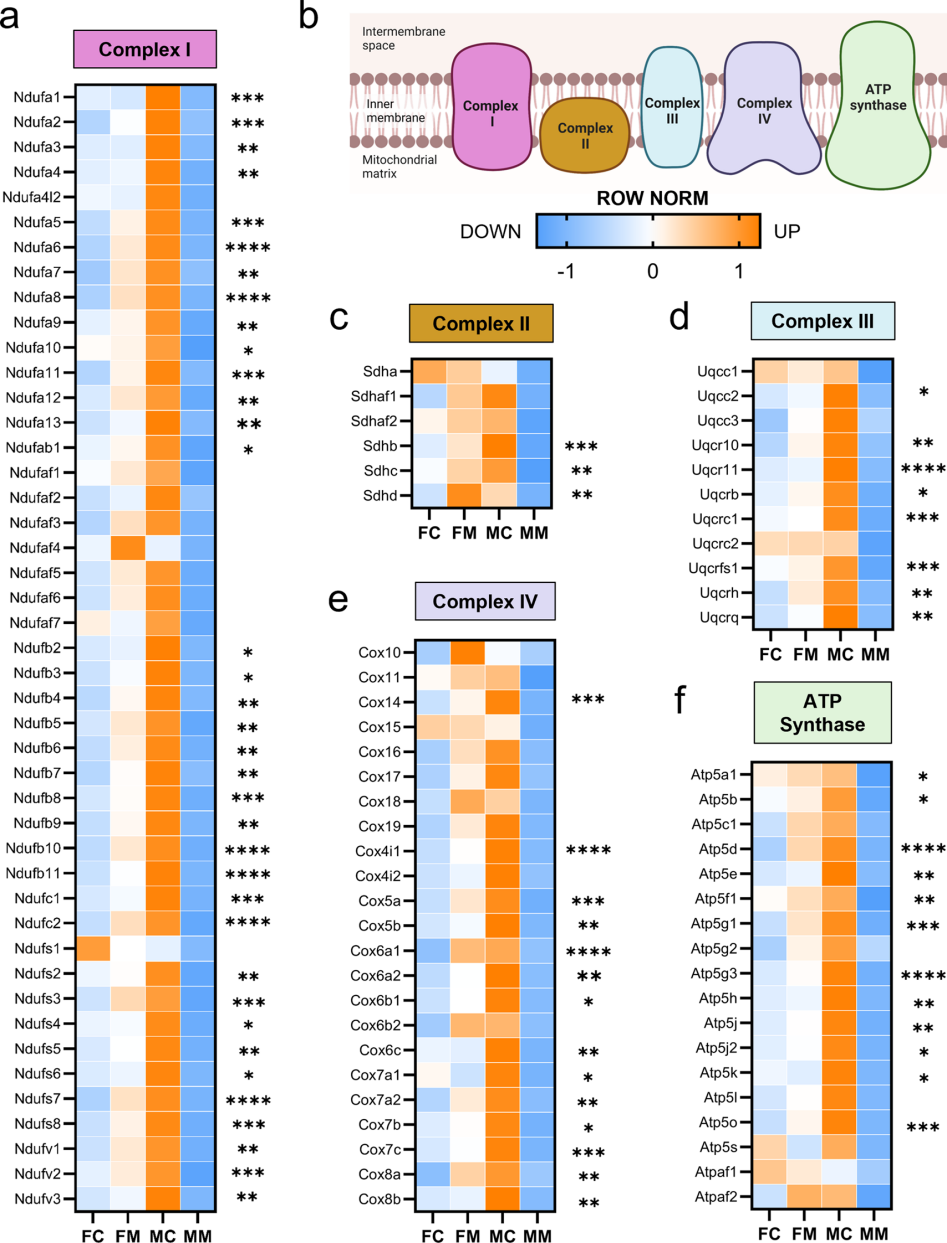
Sex differences in mitochondrial electron transport genes during myocarditis. Row normalized RPKM comparing female controls (FC), females with myocarditis (FM), male controls (MC) and males with myocarditis (MM) for nuclear encoded genes for **a**, complex I, **b**, color-coded illustration of the mitochondrial electron transport chain; **c**, complex II, **d**, complex III, **e**, complex IV, and **f**, ATP synthase. * *p <* 0.05, ** *p* < 0.01, *** *p* < 0.001, **** *p* < 0.0001

Based on our finding of sex differences in pathways related to mitochondrial respiration during myocarditis, we next focused our analysis on mitochondrial electron transport chain (ETC) genes (Fig. 8). Using reads per kilobase per million (RPKM) from RNAseq results, we compared the expression of murine nuclear encoded ETC transcripts for each complex in the ETC. We found that 36 genes out of 45 (80%) that form Complex I were significantly lower in males with myocarditis compared to females with myocarditis (Fig. 8a, b), suggesting Complex I dysfunction in males with myocarditis. Significant differences in genes comparing males to females with myocarditis are indicated by asterisks (Fig. 8). Males with myocarditis also had significant decreases in 3 genes out of 6 (50%) in Complex II (Fig. 8c), 8 genes out of 11 (73%) in Complex III (Fig. 8d), 14 genes out of 23 (61%) in Complex IV (Fig. 8e), and 12 genes out of 18 (67%) in the ATP synthase compared to females with myocarditis (Fig. 8f). These findings show that expression of nuclear encoded mitochondrial ETC transcripts increase in females during myocarditis whereas they decrease in males.

### Verification of mitochondrial dysfunction in males during myocarditis using respirometry

**Fig. 9:**
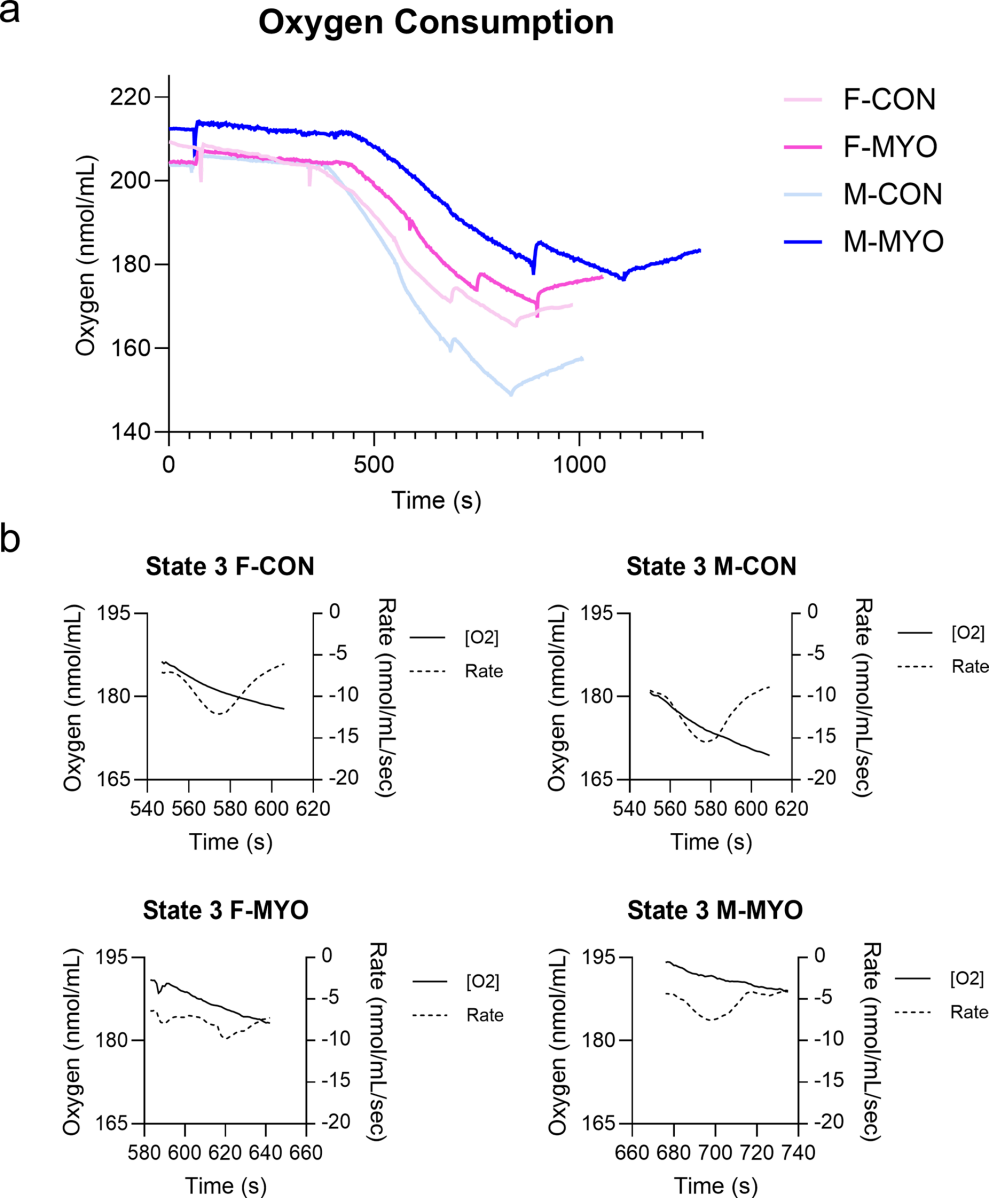
Isolated cardiac mitochondria from male mice with myocarditis have reduced oxygen consumption. Mitochondria were isolated from the heart of male (M) and female (F) BALB/c mice treated with saline (CON) or CVB3 (MYO) and harvested at day 10 pi. **a**, Representative examples of one sample from each group show mitochondrial oxygen consumption over time measured using a Clark electrode (OxyTherm). **b**, Representative examples of one sample from each group of mitochondrial oxygen consumption/concentration for State 3 are shown highlighting the slope of the solid line, which is less steep in males with myocarditis. Rate is represented by a dashed line

**Fig. 10:**
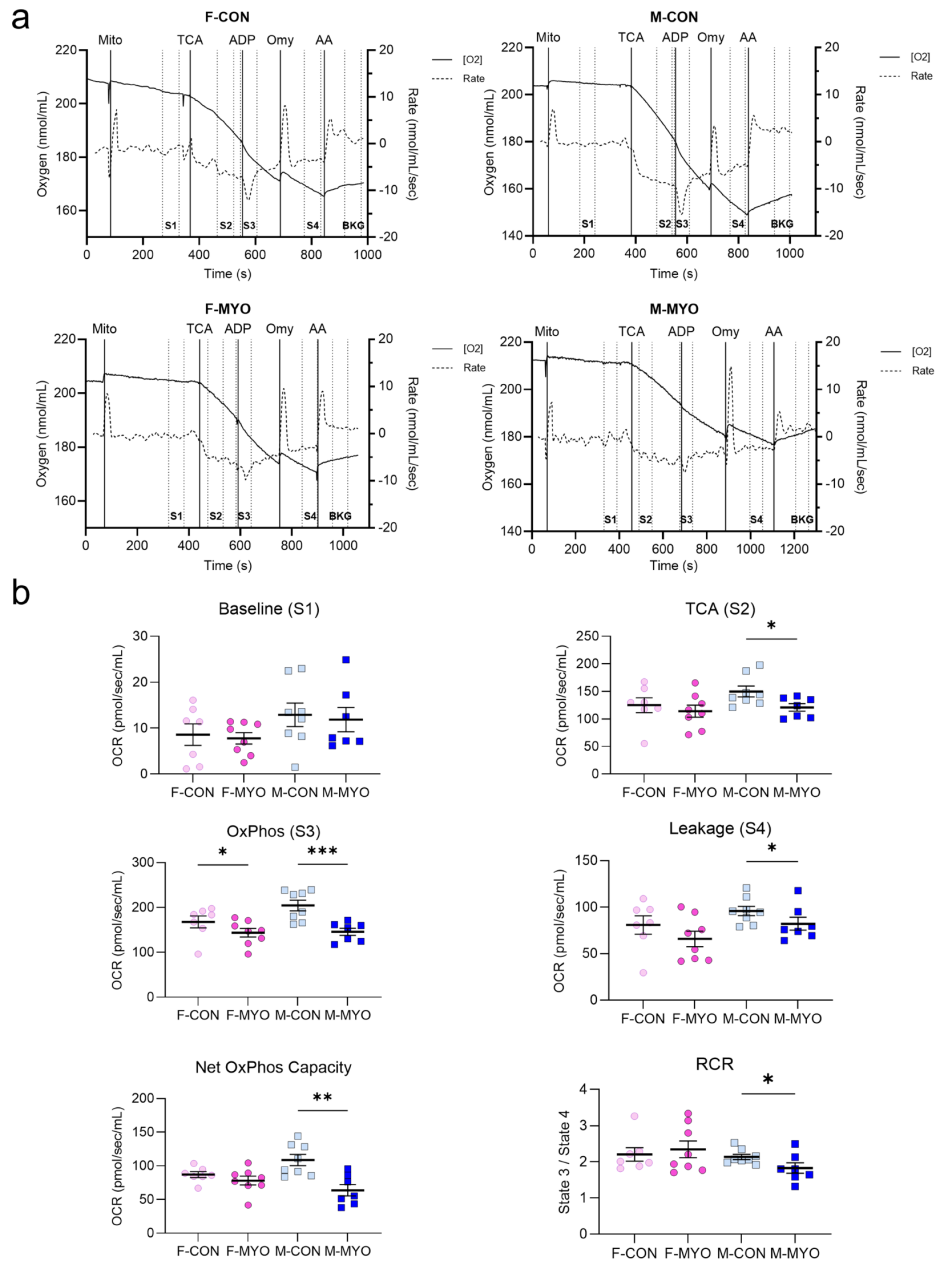
Isolated cardiac mitochondria from male mice with myocarditis have reduced oxygen consumption rate for State 2–4. Mitochondria were isolated from the heart of male (M) and female (F) BALB/c mice treated with saline (CON) or CVB3 (MYO) and harvested at day 10 pi. Mitochondrial oxygen consumption over time was measured using a Clark electrode (OxyTherm) after administration of respiratory substrate. **a**, Graphs show when mitochondria were added (Mito) followed by the administration of glutamate, malate and succinate which stimulates the tricarboxylic acid (TCA) cycle, ADP, oligomycin (Omy), and antimycin A (AA). Respiratory states were calculated after administration of the substrate and are represented by two perpendicular dotted lines with the state name inside. Background oxygen consumption rate is shown in Additional File 1: Figure S9. Solid line depicts oxygen concentration and dotted line the rate. **b**, Graphs represent the slope of the oxygen consumption rate (OCR) during the states shown in **a**. Baseline represents the OCR before addition of substrates to stimulate the TCA cycle. Oxidative phosphorylation (OxPhos) occurs after induction with ADP. Leakage represents leakage of protons through the inner mitochondrial membrane. Net OxPhos Capacity is the difference between coupled (State 3) and uncoupled (Leakage/ State 4) respiration. Respiratory control ratio (RCR) is State3/State 4 and indicates mitochondrial efficiency. Unpaired one-way Mann-Whitney tests; *p < 0.05, ** *p* < 0.01, *** *p* < 0.001

To verify our RNA sequencing findings, we examined the oxygen consumption rate of mitochondria isolated from the heart in male and female controls and during myocarditis at day 10 pi. After isolating mitochondria and validating their purity using western blot (Additional File 1: Figure S7), we measured mitochondrial respiratory capacity using a Clark electrode (Hansatech, Oxytherm + R) (Figs. 9 and 10). Representative images of the oxygen consumption rate (OCR) of mitochondria isolated from the heart during myocarditis in males and females compared to controls is shown in Fig. 9. Both males and females with myocarditis had a reduced OCR, which was more greatly reduced in males (Fig. 9a). Representative images of the slope during State 3 illustrate that males with myocarditis had a reduced OCR (Fig. 9b). Figure 10a shows representative images of OCR by group for all states after administration of substrates and inhibitors. Quantitated data at each state are shown in Fig. 10b. At baseline, during State 1 respiration, there were no significant differences in the OCR by sex between mitochondria for controls or mice with myocarditis. We found a decrease in the OCR during State 2 after administering glutamate, malate, and succinate (TCA) in males with myocarditis compared to controls (*p* = 0.027), but not females (*p* = 0.168) (Fig. 10b). In the presence of ADP (State 3), both females (*p* = 0.0361) and males (*p* = 0.0006) with myocarditis had a significant decrease in OCR, but the difference between medians in males was greater than females (53.64 pmol/sec/mL for males compared to 35.25 pmol/sec/mL for females). State 4 respiration (Leakage) was induced with oligomycin and was significantly decreased in males with myocarditis (*p* = 0.0361), but not females (*p* = 0.141) compared to controls. We found the net oxidative capacity, which is the difference between State 3 and State 4, were significantly decreased in males (*p* = 0.0047), but not females (*p* = 0.198). The respiratory coupled ratio (RCR), which is a measure of mitochondrial efficiency, was also significantly decreased in males with myocarditis (*p* = 0.0361), but not females (*p* = 0.433) (Fig. 10b). Background respiration was not significantly different between any of the groups (Additional File 1: Figure S8). Thus, the respirometry data verify the RNA-sequencing findings; females regulate mitochondrial homeostasis better than males during viral myocarditis by both measures of oxidative capacity and efficiency.

### ERRα identified using TRANSFAC as a candidate transcription factor that may regulate mitochondrial genes

**Fig. 11:**
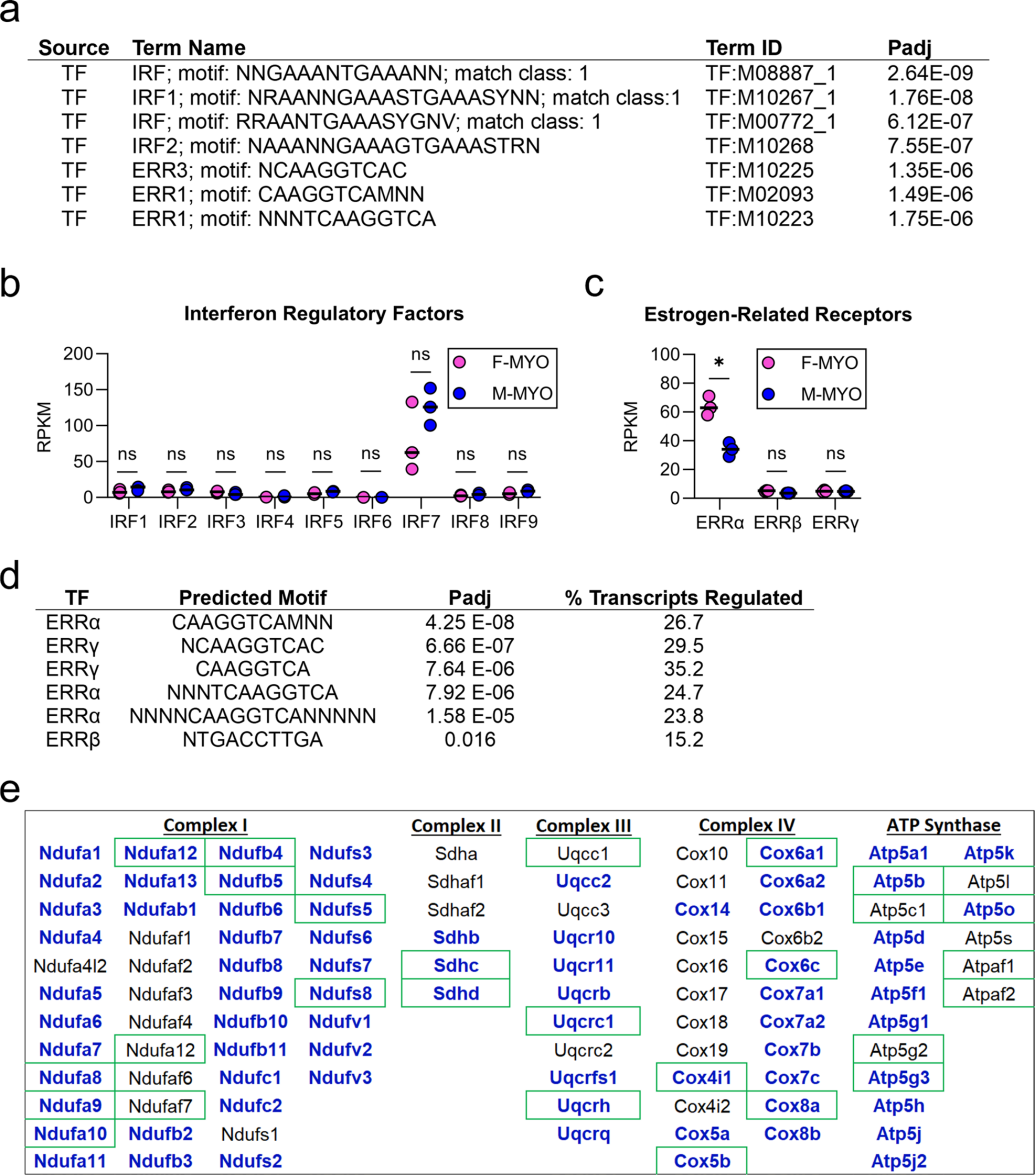
TRANSFAC analysis identifies interferon regulatory factors and estrogen-related receptors as potential mediators of sex difference during myocarditis. **a**, TRANSFAC results comparing females with myocarditis (F-MYO) and males with myocarditis (M-MYO); RPKM (reads per kilobase per million) using false discovery rate to compare F-MYO and M-MYO for **b**, interferon regulatory factors (IRFs) and **c**, estrogen-related receptors (ERRs); **d**, predicted binding capacity of ERRs for electron transport chain transcripts; **e**, significantly different transcripts by sex in electron transport genes are indicated by bold blue lettering, green boxes indicate genes that ERRα predicted to bind to

To identify transcriptional regulators that might globally affect the major changes in transcripts according to sex that we observed, we used the gProfiler “TRANScription FACtor database” (TRANSFAC) as an unbiased approach to identify candidate transcription factors. TRANSFAC analysis identified interferon regulatory factors (IRFs) and estrogen-related receptors (ERRs) as the top potential transcriptional regulators of gene differences between males and females with myocarditis (Fig. 11a). We compared expression of all nine IRFs (Fig. 11b) and all three ERRs (Fig. 11c) from RNA sequencing data and found that none of the nine IRFs were significantly different by sex but ERRα was significantly higher in females with myocarditis compared to males (FDR = 0.03).

We assessed the predicted binding capacity of ERRs among the nuclear encoded mitochondrial respiratory chain complexes examined in Fig. 8 using TRANSFAC. ERRα and ERRγ shared a core predicted binding motif of TCAAGGTCA with ERRα present in the proximal promoter of around 30% of the nuclear encoded mitochondrial respiratory chain complex transcripts (Fig. 11d). This is in line with a previous study that showed that both ERRα and ERRγ target a common set of promoters involved in mitochondrial respiration and ATP production in the hearts of wild type male mice [22]. Indeed, respiratory chain complex genes that were predicted to be bound by ERRα (or ERRγ) are indicated by green boxes and those that were significantly different by sex in Fig. 8 are indicated with bold blue lettering in Fig. 11e. These findings suggest that ERRs may influence the global sex differences in mitochondrial gene expression that were observed during myocarditis.

### Females upregulate master regulators of mitochondrial homeostasis during myocarditis

Next, we verified whether sex differences existed in global transcriptional regulators of mitochondrial metabolism including PPARγ co-activator alpha (PGC1α), nuclear respiratory factor 1 (NRF1) and ERRα. Using qRT-PCR, we found that PGC1α transcript levels were significantly increased in the heart during myocarditis when males and females with myocarditis were combined compared to controls (*p* < 0.0001) (Additional File 1: Figure S9a) or examined individually by sex compared to controls (females *p* < 0.0001, males *p* = 0.0003) (Additional File 1: Figure S9b). Comparing males to females with myocarditis, females with myocarditis had significantly higher levels of PGC1α in the heart compared to males (*p* = 0.0458) (Additional File 1: Figure S9b).

PGC1α interacts with NRF1 leading to transcription of mitochondrial genes including ATP synthase, cytochrome-c, cytochrome-c-oxidase subunit IV, and mitochondrial transcription factor A which activates mitochondrial DNA replication and transcription [23–25]. We found that NRF1 RNA levels were significantly decreased when males and females with myocarditis were combined compared to controls (*p* < 0.0001) (Additional File 1: Figure S9c). This was also observed when NRF1 levels were examined in males with myocarditis versus controls (*p* < 0.0001) or females with myocarditis versus controls (*p* < 0.0001) (Additional File 1: Figure S9d). However, NRF1 levels in the heart during myocarditis were significantly higher in females compared to males (*p* = 0.0315) (Additional File 1: Figure S9d).

**Fig. 12:**
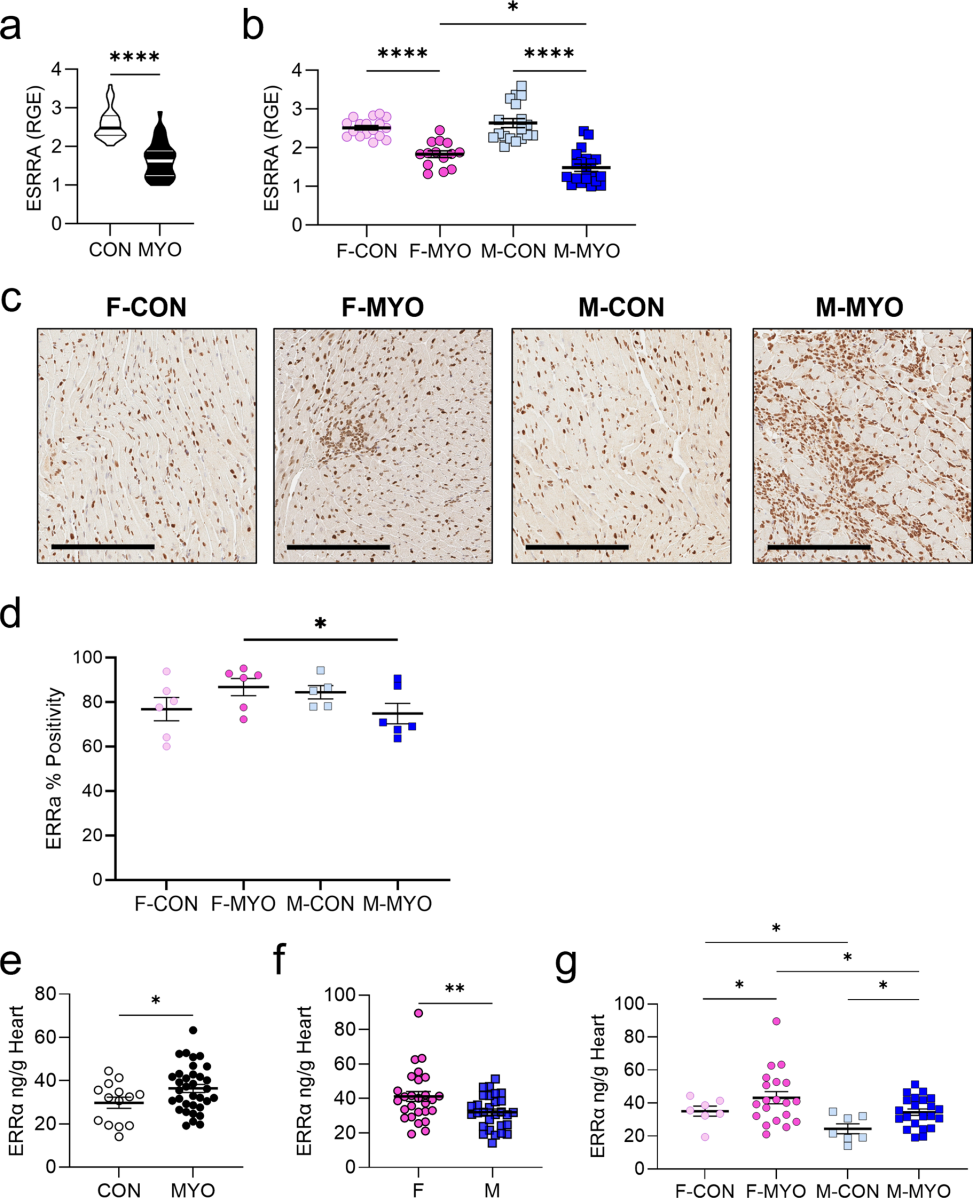
Females with myocarditis express higher levels of cardiac ERRα transcript and protein compared to males. **a**, **b**, Relative gene expression (RGE) of ESRRA/ ERRα relative to the housekeeping gene HPRT for females (F) or males and (M) controls (CON) or myocarditis (MYO): F-CON (*n =* 15), F-MYO (*n =* 20–21), M-CON (*n =* 17–18), and M-MYO (*n =* 19–20). **c**, Representative heart sections of IHC for ERRα, Scale bars = 200 μm. **d**, Quantification of IHC using Image J. **e**, **f**, **g**, ERRα ELISA from whole heart homogenate supernatant comparing **e**, CON (*n* = 14) to MYO (*n* = 42); **f**, all females (F, *n =* 27) to all males (M, *n* = 29) regardless of disease state; **g**, two-way ANOVA of F-CON (*n* = 7), F-MYO (*n* = 20), M-CON (*n* = 7), and M-MYO (*n* = 22) found effect of sex (*p* = 0.006) and myocarditis (*p* = 0.017). *p < 0.05, ** *p* < 0.01, **** *p* < 0.0001

We also assessed ERRα (ESRRA mRNA) by sex, which had previously been identified using TRANSFAC (Fig. 11). ERRα is designated as an orphan nuclear receptor [26–28] but recent evidence suggests its endogenous ligand may be cholesterol [29–31]. ERRα displays some basal activity during nominal cell states but transcriptional activity is enhanced by co-activator interaction with PGC1α [25]. PGC1α is a transcriptional co-activator protein that binds ERRα (not as a ligand but as a co-factor [32]) and promotes its transcriptional activity [26]. ERRα-PGC1α have been found to regulate hundreds of genes involved in mitochondrial oxidative phosphorylation, the tricarboxylic acid (TCA) cycle, fatty acid beta-oxidation, and glucose and lipid metabolism [26, 27, 33–36]. When we examined ESRRA mRNA levels by qRT-PCR during myocarditis, we found that ESRRA was significantly decreased in mice with myocarditis compared to controls (*p* < 0.0001) (Fig. 12a). This was also true when we examined females with myocarditis compared to controls (*p* < 0.0001) or males with myocarditis compared to controls (*p* < 0.0001) (Fig. 12b). Similar to PGC1α and NRF1, we found that females had significantly higher levels of ESRRA transcript during myocarditis compared to males (*p* = 0.0128) (Fig. 12b).

To examine cardiac protein expression of ERRα in the murine heart, we performed immunohistochemistry (IHC). Representative images show that ERRα staining intensity was high for both cardiomyocyte and immune cell nuclei (Fig. 12c). Quantifying the IHC, we did not observe a significant difference between ERRα levels in male vs. female controls (*p* = 0.247) or comparing female controls to females with myocarditis (*p* = 0.310) or male controls vs. males with myocarditis (*p* = 0.247) (Fig. 12d). However, we found significantly higher levels of ERRα expression in females with myocarditis compared to males with myocarditis (*p* = 0.0260) (Fig. 12d), like RNA findings.

To further verify ERRα transcript findings during myocarditis we examined heart protein levels of ERRα by ELISA. We found that ERRα was significantly increased when males and females with myocarditis were combined compared to controls (*p* = 0.0486) (Fig. 12e) and in all females compared to all males regardless of disease status (*p* = 0.0094) (Fig. 12f). To determine the effect of sex versus myocarditis in ERRα protein expression, we performed two-way ANOVA and found a significant effect of sex (*p* = 0.006) and myocarditis (*p* = 0.017), indicating sex differences drove the main effect (Fig. 12g). We also found that ERRα protein levels were significantly increased in females with myocarditis compared to controls (*p* = 0.0340) and in males with myocarditis compared to controls (*p* = 0.0340) (Fig. 12g). Importantly, ERRα protein levels were significantly increased in females with myocarditis compared to males with myocarditis (*p* = 0.0234) (Fig. 12g), confirming qPCR findings (Fig. 12b). Thus, by all methods we examined, ERRα transcript and protein levels were elevated in the heart of females compared to males during myocarditis.

### ChIP sequencing confirms binding of ERRα to mitochondrial genes, but only in females with myocarditis

**Fig. 13:**
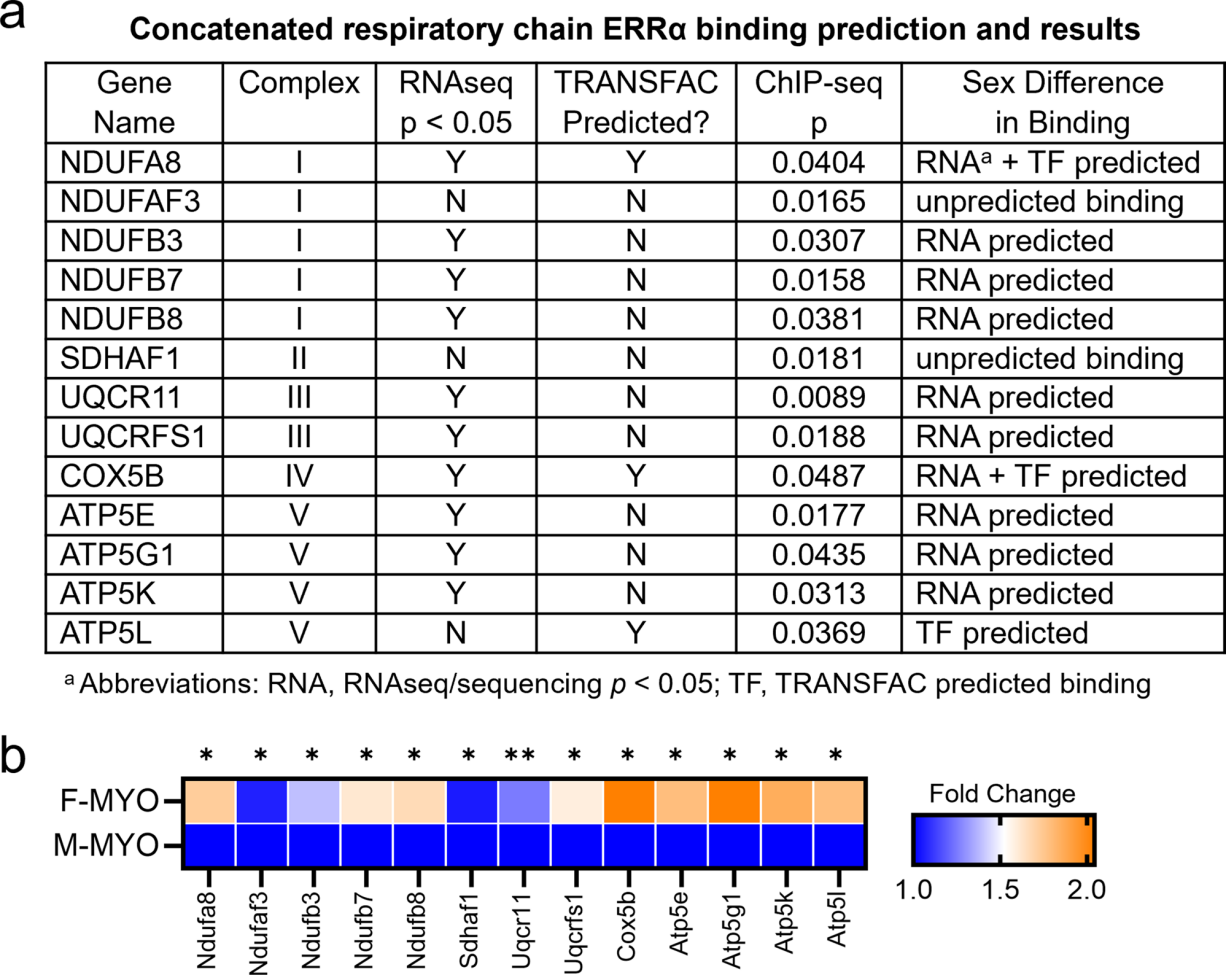
ChIP sequencing confirms binding of ERRα to mitochondrial genes, but only in females with myocarditis. **a**, The table compares ChIPseq findings to the results obtained by RNAseq and TRANSFAC. **b**, Heatmap depicting mitochondrial ETC genes bound by ERRα using ChIP (blue, negative; orange, positive) comparing females (F) with myocarditis (MYO) to males (M) with myocarditis. *p < 0.05, ** *p* < 0.01

To determine whether the transcription factor ERRα binds to mitochondrial ETC genes during myocarditis, we conducted chromatin immunoprecipitation (ChIP) sequencing (ChIPseq) comparing ERRα binding to ETC genes in the heart of male and female mice with myocarditis. We found that ERRα bound significantly to regions in genes found in complex I-IV of the ETC (Fig. 13a, data found in Additional File 2). Figure 13a compares ChIPseq findings to the results obtained by RNAseq and TRANSFAC. All the nuclear encoded mitochondrial ETC regions with higher ERRα binding occurred in females with myocarditis, and no significant binding was found in males with myocarditis (Fig. 13a). In females, 5 genes from complex I were identified including *Ndufa8* (*p* = 0.040), *Ndufaf3* (*p* = 0.017), *Ndufb3* (*p* = 0.031), *Ndufb7* (*p* = 0.016), and *Ndufb8* (*p* = 0.038) (Fig. 13b). One region of binding occurred for the complex II subunit *Sdhaf1* (*p* = 0.018), and two from complex III which included *Uqcr11* (*p* = 0.009) and *Uqcrfs1* (*p* = 0.019). *Cox5b* (*p* = 0.049) was the sole significantly bound nuclear encoded mitochondrial ETC region from complex IV, and in complex V (i.e., the ATP synthase) *Atp5e* (*p* = 0.018), *Atp5g1* (*p* = 0.044), *Atp5k* (*p* = 0.031), and *Atp5l* (*p* = 0.037) bound more in females with myocarditis compared to males (Fig. 13). Only two genes (*Ndufa8* and *Cox5b*) were dually predicted to have higher ERRα binding from RNAseq and TRANSFAC analyses (Fig. 13a). Interestingly, ERRα bound *Ndufaf3* and *Sdhaf1* more significantly in females with myocarditis compared to males with no prediction for these genes by RNAseq or TRANSFAC. The remaining significantly bound nuclear encoded mitochondrial ETC regions were predicted by RNAseq alone (Fig. 13a). These results provide evidence that ERRα binds to genes of the mitochondrial ETC of females during myocarditis but not males and may be responsible, at least in part, for the improved mitochondrial homeostasis observed in females during myocarditis.

## Discussion

In this study we show for the first time that male mice with CVB3 myocarditis have reduced mitochondrial transcription and function compared to females using an autoimmune model of CVB3 myocarditis that is highly translational to human disease [37]. We show that females with myocarditis have higher expression of several master regulators of mitochondrial homeostasis including PGC1α, NRF1 and ERRα compared to males. Females, who have less severe myocarditis, recovered mitochondrial homeostasis during myocarditis while mitochondrial function worsened for males. At baseline in controls, males had higher mitochondrial gene expression than females, which may be explained by a higher energetic demand in males because of larger hearts. A sex-specific effect of ERRα on ETC genes suggests a potential regulatory mechanism for our observed sex differences in mitochondrial gene transcription. ChIPseq analysis verified that ERRα bound mitochondrial genes in a sex-specific manner during viral myocarditis.

A study by Dufour et al. using ERRα deficient mice, found that when ERRα was low in the heart PGC1α was elevated as a compensation mechanism [22]. PGC1α was originally identified as a regulator of mitochondrial function in brown adipose tissue but was later also found to be expressed at high levels in cardiac tissue where it influences cardiovascular health and disease [26, 38]. PGC1α globally regulates mitochondrial pathways in response to stresses such as cold, fasting and infection [36, 39–42]. Thus, the metabolic stress of CVB3 infection is a likely explanation for the elevated levels of PGC1α that we observed in males and females with myocarditis compared to controls.

ERRα has been found to be critical in regulating mitochondrial homeostasis in the heart demonstrated by Dufour et al. using male ERRα deficient C57BL/6 mice [22]. They found that ERRα targeted mitochondrial NRF1, cyclic AMP-response element binding protein (CREB), and STAT3 [43]. Surprisingly, we observed an inverse relationship between mRNA and protein levels of ERRα in the heart with increased levels by ELISA in males and females with myocarditis. Regardless of mRNA levels, ERRα protein levels can be highly regulated by post-translational modifications and metabolic stress [44]. Previously, it was notably shown that under specific cellular metabolic stress conditions, such as reactive oxygen species (ROS) exposure, ERRα protein levels can be dramatically altered in a proteasome-dependent manner [44]. Similarly, insulin or glucose stimulation increased ERRα protein levels without altering mRNA expression in hepatocytes [45], further strengthening the hypothesis that ERRα protein levels can be altered under metabolic pressure independently of gene expression. Indeed, transcription factor expression often does not necessarily provide detailed information as to the direct actions of that transcription factor; and in this case, how ERRα activity may differ by sex during myocarditis. For this reason, we conducted ChIP sequencing of ERRα to determine binding activity. We found that ERRα primarily binds mitochondrial regions of the ETC, but only in female mice with myocarditis. These findings suggest that ERRα plays a protective role in females with myocarditis by improving mitochondrial homeostasis, but no protection in males because of a lack of binding to ETC genes. We are currently further characterizing the genomic interactions of ERRα to elucidate its sex-specific transcriptional activity during viral myocarditis.

Interleukin (IL)-1α, IL-1β, and tumor necrosis factor (TNF)α are known to activate the transcriptional activity of PGC1α through direct phosphorylation of p38 mitogen-activated protein (MAP) kinase [25, 46]. We found previously that IL-1β levels are increased in the heart of males with myocarditis, while cardiac levels of TNFα are increased in females in our CVB3 model of myocarditis [10, 47]. Most cardiac inflammatory cells during acute myocarditis at day 10 pi are CD11b+ (macrophages and mast cells) that express TLR4 and release IL-1β [16]. We showed previously that elevated IL-1β levels in the heart directly correlate to elevated cardiac inflammation in males with myocarditis and poor cardiac function by echocardiography [10]. Importantly, Remels et al. showed that elevated TNFα levels in cardiomyocytes in culture following CVB3 infection were directly associated with decreased PGC1α mRNA levels [48].

Additional evidence of the negative effect that IL-1β can have on mitochondrial gene expression in males was found in studies by Ge et al. [19, 49, 50]. Calpain is a calcium-dependent protease that facilitates apoptotic signaling and localizes to the mitochondria during CVB3 infection to proteolyze mitochondrial substrates, leading to increased mitochondrial fission (mitochondrial fragmentation due to pathological or physiological stress). Inhibition of calpain reduced mitochondrial fission and cardiomyocyte apoptosis during myocarditis [49]. Liu et al. showed that mitochondrial calpain-1 induces mitochondrial dysfunction and ROS production which activates the NLRP3 inflammasome, which leads to IL-1β production [50]. Macrophages, which are the predominant infiltrating immune cells during myocarditis, were found to respond to CVB3 infection by upregulation of calpain-4; RNA sequencing of CVB3 infected macrophages in vitro revealed predominant enrichment for pathways related to macrophage maturation and interleukin signaling, and loss of calpain-4 reduced IL-1β expression [19]. Although we did not specifically examine IL-1β in this study, our previous findings may be relevant to the current results that suggest that elevated inflammatory cells and cytokines, especially IL-1β, in the heart of males during acute CVB3 myocarditis [10] may directly contribute to lower PGC1α levels in males than females leading to decreased mitochondrial gene expression in the heart at that timepoint.

In general, sex differences are known to exist in mitochondrial bioenergetics [51, 52], but we provide sex-specific information in the context of viral myocarditis. Similar to previous studies that examined gene changes in the heart during CVB3 myocarditis in male mice [10, 48, 53], we found that the predominant gene expression changes aside from immune pathways were mitochondrial genes. Previously, Remels et al. reported that PGC1α mRNA and NRF1 protein levels were significantly decreased in the heart of male mice with CVB3 myocarditis compared to controls from day 4 to 7 pi [48]. They also found decreased gene expression profiles for ETC genes during myocarditis in males [48], similar to our results, but they did not examine females with myocarditis.

Ebermann et al. also examined gene expression in males with CVB3 myocarditis comparing C57BL/6 (B6) to A.SW/SnJ mice [54]. They used a tissue culture CVB3-induced model that produces similar inflammation in these two strains of mice but different cytokine profiles [54]. This tissue-culture CVB3 model produces a completely different myocardial immune profile than our model of autoimmune CVB3-myocarditis comparing BALB/c to B6 mice [9, 55, 56]. However, Ebermann et al. found that A.Sw/SnJ male mice with myocarditis have significantly lower ETC gene expression compared to controls that was directly related to the level of viral replication in the heart [54]. In our model of CVB3 myocarditis there are no sex differences in VP1 RNA levels or viral replication based on plaque assay during acute myocarditis [16]. The findings of Ebermann et al. may reflect, however, the finding of Sin et al. who showed in cultured cardiomyocytes that CVB3 localizes to mitochondria, induces mitophagy, and disseminates from the cell in an extracellular autophagosome-bound virus-laden mitochondrial complex [57]. Sin et al. showed that upstream suppression of the mitophagy pathway in HL-1 cardiomyocytes using small interfering RNA (siRNA) targeted to dynamin-related protien-1 (DRP1) or mitochondrial division inhibitor (Mdivi-1) significantly reduced virus production from cardiomyocytes [57] (as mitochondrial fission is an early stage of mitophagy). Other viruses that cause myocarditis such as human immunodeficiency virus (HIV), hepatitis B and C, influenza, Epstein-Barr virus and SARS-CoV-2 have been found to localize to mitochondria and hijack aspects of the mitochondrial machinery for replication [5, 58–60]. This might explain why so many diverse viruses without specific tropism for cardiac tissue (i.e., murine cytomegalovirus/ MCMV, SARS-CoV-2, CVB3) are able to cause myocarditis, since they can target a mitochondria-rich environment for a replication advantage.

TRANSFAC analysis identified IRFs and ERRs as key transcription factors that could mediate sex differences in gene expression in our model. CVB3 infection strongly activates type I interferons (IFNαs and IFNβ) and type II (IFNγ) IFN production during myocarditis to reduce viral replication via Toll-like receptor (TLR) activation including TLR3, TLR4, TLR7 and TLR9 and the transcription factor TIR domain-containing adaptor inducing interferon-β (TRIF) which is downstream of TLR3 and TLR4 [10, 47, 61–63]. Although IFNγ is increased in our model of CVB3 myocarditis in males [16, 18], we showed that elevated IFN levels in male BALB/c mice with myocarditis are mediated by IL-18, which is downstream from TLR4, rather than traditional STAT4/IL-12 transcriptional activity [18, 64]. We do not observe a sex difference in viral levels in the heart during myocarditis in our CVB3 mouse model and sex differences in IFNγ are not mediated by classic IFN signaling. Therefore, it is not surprising that we did not observe a significant difference by sex of the nine IFN transcription factors (Fig. 11b).

Sex hormones are known to strongly drive the innate and adaptive immune response to infections in general and during myocarditis [8, 65, 66], and to confer sex differences in mitochondrial morphology and function via estrogen receptor (ER) nuclear and mitochondrial transcription factor activity [40, 67, 68]. The heart of females is known to have greater mitochondrial efficiency, fatty acid utilization during exercise, and calcium retention whereas males have more mitochondrial content, reactive oxygen species production, and higher calcium uptake rate for example [40, 67]. A summary of these known sex differences in mitochondrial-related genes and pathways can be found in Additional File 1: Table S2. ERRα was originally named based on its sequence homology to ERα [69]. Although sex differences in some mitochondrial gene expression pathways during CVB3 myocarditis may be explained by sex hormones, specifically estrogen via ERs, 17β-estradiol and other natural estrogens are not endogenous ligands for ERRα [69, 70]. Two groups have provided evidence that support the hypothesis that cholesterol is the endogenous ligand for ERRα with in vitro and in vivo data [29–31]. During nominal cellular states and unbound by its ligand, ERRα displays some transcriptional activity [26, 33, 71]. Based on structural homology, ERRs are speculated to share target genes, coregulatory proteins, and sites of action with ERs and therefore actively influence the estrogenic response [72]. The genotype-tissue expression (GTEx) project identified ERRα as a “sex-biased” transcriptional regulator in humans [73]. Overall, this could explain sex differences in ERRα expression.

Lee et al. found sex differences in ERRα levels in the brains of 4-week-old immature female but not male mice that had been treated with a chemical known to reduce mitochondrial function [74], suggesting sex differences in ERRα function prior to the production of circulating hormone production. De Jesus-Cortez et al. found that ERRα deficient adult female mice had defects in neural function in a mouse model of eating disorders, which mainly affect women, which was not observed in ERRα deficient male mice, and they concluded that ERRα was required for optimal mitochondrial function in females [75]. Watson et al. found sex-specific effects of ERRα expression in the hearts of female but not male mice in a model of heart failure [76]. We are the first to report sex differences in ERRα expression in the hearts of healthy mice and mice with viral myocarditis. Subsequent studies are needed to further characterize sex-specific effects of ERRα on mitochondrial function during CVB3 myocarditis. We are currently conducting further analysis of sex differences in gene-specific transcription factor-DNA interaction of ERRα using ChIP in viral myocarditis.

### Perspectives and significance

In this study we show for the first time that males with CVB3 myocarditis have reduced mitochondrial gene expression of nuclear-encoded ETC genes compared to females. Females had higher levels of global regulators of mitochondrial function compared to males which may promote mitochondrial homeostasis that protects females from cardiac damage following infection and inflammation. ERRα is a strong candidate to mediate these sex differences, at least in part, and should be further characterized.

## Methods

### Myocarditis model

Male and female 6–8 week-old BALB/cJ mice (stock# 651) were obtained from Jackson Laboratory (Bar Harbor, ME). Mice were inoculated with sterile phosphate buffered saline (PBS) (vehicle control) or 10^3^ plaque forming units (PFU) of heart-passaged CVB3 intraperitoneally (ip) on day 0 and hearts collected on day 10 pi, as previously described [77]. This is an autoimmune model of myocarditis using live virus as the adjuvant that closely resembles experimental autoimmune myocarditis and human lymphocytic myocarditis (reviewed in [55, 56]). The Nancy strain of CVB3 was originally obtained from the American Type Culture Collection (ATCC; Manassas, VA) and grown in Vero cells (ATCC), to create a tissue culture-derived virus stock as previously described [77]. Briefly, 100µL of tissue culture virus (10^3^ PFU) was injected ip into 4-week-old female BALB/c mice and virus obtained from hearts at day 3 pi by homogenization in Gibco Minimum Essential Media (Thermo-Scientific, Waltham, MA, 11095-080) supplemented with 2% heat inactivated FBS. Homogenized hearts were centrifuged at 4 C for 20 min at 795*g*. Homogenized supernatant that contains infectious virus and damaged heart proteins (heart-passaged virus) was stored at -80 C until used to induce myocarditis, as described in [77]. We used 7–11 mice/group in individual experiments and pooled data for figures. Not all endpoints can be obtained from each experiment so the number per group varies in individual figures. We showed previously that at least 7 mice/group are needed to detect sex differences in myocarditis [16].

### Histology

Mouse hearts were cut longitudinally and fixed in 10% phosphate-buffered formalin and embedded in paraffin for histological analysis. 5 μm sections were stained with hematoxylin and eosin (H&E) to detect inflammation. Myocarditis was assessed as the percentage of the heart with inflammation compared to the overall size of the heart section using a microscope eyepiece grid, as previously [9, 78]. Sections were scored by two individuals blinded to the treatment group.

### Quantitative real time PCR

RNA was isolated from mouse hearts using Qiagen’s Fibrous Tissue Mini Kit (Qiagen 74704) before the concentration (Abs. 260) and quality (Abs. 260/280) of preparations were assessed using a Nanodrop. cDNA was generated using the iScript cDNA synthesis kit (Biorad, #1708891). Quantitative real time PCR (qRT-PCR) was assessed with Taqman probes (CD45/*Ptprc* Mm01293577_m1; CD11b/*Itgam* Mm00434455_m1; F4/80/*Adgre1* Mm00802529_m1; peroxisome proliferator-activated receptor gamma coactivator 1 (PGC1α)/*Ppargc1a* Mm01208835_m1; nuclear respiratory factor 1 (NRF1)/*Nrf1* Mm01135606_m1; estrogen-related receptor-α (ERRα)/*Esrra* Mm00433143_m1) and normalized against hypoxanthine phosphoribosyltransferase 1 (HPRT) (*Hprt*, Mm03024075_m1) to determine relative gene expression (RGE) using ΔΔCt as previously [78, 79].

### RNA sequencing

At the time of harvest, half of the heart was collected for histological evaluation using H&E to determine the severity of myocardial inflammation and the other half was snap frozen in liquid nitrogen. Histology shown in Fig. 1 is combined data from 3 separate experiments. The investigator selected 3 histologically representative samples of the overall dataset from a single experiment (white symbols, *n* = 3/group) and sent to the Mayo Clinic Genome Analysis Core for library preparation and bulk-tissue RNA sequencing. Libraries for this study were prepared using the Core’s standard mRNAseq prep which uses poly A selection. RNA libraries were prepared using 200 ng of total RNA according to the manufacturer’s instructions for the TruSeq Stranded mRNA Sample Prep Kit (Illumina, San Diego, CA). The concentration and size distribution of the completed libraries was determined using an Agilent Bioanalyzer DNA 1000 chip (Santa Clara, CA) and Qubit fluorometry (Invitrogen, Carlsbad, CA). Libraries were sequenced at 50 million fragment reads per sample following Illumina’s standard protocol using the Illumina cBot and HiSeq 3000/4000 PE Cluster Kit. The flow cells were sequenced as 100 × 2 paired end reads on an Illumina HiSeq 4000 using HiSeq 3000/4000 sequencing kit and HiSeq Control Software HD 3.4.0.38 collection software. Base-calling was performed using Illumina’s RTA version 2.7.7.

### RNA sequencing analysis

After next-generation RNA sequencing, the Mayo Clinic Genome Analysis Core provided differential expression data. We compared PBS control females vs. females with myocarditis, PBS control males vs. males with myocarditis, and females with myocarditis vs. males with myocarditis. Because of small group size, we assessed intra-group variability using *ClustVis* [80] by performing unsupervised hierarchical clustering using Euclidean row and column distances and principal component analysis (PCA).

Differential expression analysis was performed by the Mayo Clinic Genome Analysis Core and gene names were converted to murine ensemble IDs (ENSMUSG) for analysis. We performed enrichment analysis as in Reimand et al. [81]. For gProfiler, transcripts with nominal p-value < 0.05 were ordered from most to least significant and an ordered query was run (gene sets with 5-350 entities were included). At the time of analysis, we excluded duplicate transcripts or those not recognized by gProfiler. The same set of transcripts used for gProfiler were ordered by logFC to perform gene set enrichment analysis (GSEA) pre-ranked utilizing a combined gene matrix transposed (GMT) from gProfiler. GSEA pre-ranked was performed with default gene set size restriction (15–500) and permutation parameters (1,000).

GSEA results were plotted in Cytoscape (Version 3.7.23) using *Enrichment Map* with a node (i.e., gene set/pathway) cutoff of FDR(Q) value < 0.1 and edge cutoff of 0.375. Nodes were clustered based on shared genes and *AutoAnnotate* was used to identify clusters of nodes (sometimes referred to as “super-clusters” in this text). We selected all nodes to create a combined heat map of the top 273 genes and additionally selected mitochondrial-related nodes to create a combined heat map of the top 132 mitochondrial genes by group (row-normalized by Cytoscape). Combined heat maps for top NES pathways in F-CON vs. F-MYO and M-CON vs. M-MYO comparisons and mitochondrial pathways in F-MYO vs. M-MYO comparison (including the combined auto-annotated cluster of gene sets “respiratory complex mitochondrial) were generated from Cytoscape (row-normalized). For Metascape enrichment analysis, we excluded all genes with p > 0.05, and ran corresponding gene sets for each phenotype with the *Express Analysis* option (Metascape.org) [17] for the following comparisons: F-CON vs. F-MYO, M-CON vs. M-MYO, and F-MYO vs. M-MYO. We averaged MCODE clusters’ Log10 p values (rounded to nearest whole number) to obtain values listed in Figs. 5c and 6c.

A list of the murine nuclear encoded mitochondrial respiratory chain transcripts was generated from the Mouse Genome Informatics (MGI) database from Jackson Laboratories for respiratory chain (https://www.informatics.jax.org/go/term/GO:0005746*)* and the ATP synthase (https://www.informatics.jax.org/go/term/GO:0005753). Duplicates were removed and only transcripts of the mitochondrial respiratory chain were included. Transcripts and related data were used to create a combined gene expression matrix for each complex with row normalization (using the STANDARDIZE function in Excel). Transcripts not expressed across all four groups (PBS control females, PBS control males, myocarditis females, and myocarditis males) were excluded. The final list of transcripts was used to determine percent of transcripts predicted to be regulated via estrogen related receptors using TRANSFAC in gProfiler.

### Immunohistochemistry

Heart sections (5 μm) were stained with ERRα (1:1,000, cat# PA5-28749 ThermoFisher/Invitrogen). An Envision + anti-rabbit labeled polymer (K4003) and rat-on-rodent kit (cat# RT517, Biocare, Pacheco, CA) were used as secondary antibodies for rat antibodies. Stained slides were scanned and analyzed using an Aperio AT2 slide scanner (Leica, Wetzlar, Germany). Ventricles of cardiac sections were manually selected and the default “positive pixel” algorithm adjusted for the Color Saturation Threshold to ensure the program’s accurate selection of positive and negative pixels. The hue for all algorithms used was 0.1, brown. Stain positivity (% positive) for each marker was determined with a “positivity” parameter (positivity = number of positive pixels/(number of positive + number of negative pixels)), which is normalized to the size of the heart.

### ELISA

Frozen hearts were rapidly thawed and weighed to obtain tissue wet weight before homogenizing using a polytron homogenizer in minimum essential media (MEM) with 2% fetal bovine serum (FBS). Homogenized tissue was centrifuged at 3,000 rpm at 6^°^C for 20 min and the supernatant was collected for analysis. Whole heart ERRα protein expression was quantified using the Mouse Estrogen-Related Receptor Alpha ELISA Kit from MyBioSource (cat# MBS080310, San Diego, CA). Absorbance was used to calculate concentration relative to a standard curve and normalized to tissue wet weight, as previously [47, 78, 79, 82]. The lowest detection limit for the ERRα kit was 0.1 ng/mL with a detection range of 0.25-8 ng/mL.

### Western blotting

Western blotting was conducted on mitochondrial and cytosolic fractions to validate the purity of mitochondria isolated from the heart of mice during myocarditis. Western blot preparations were obtained by taking 100 µg of protein, calculated using the Bradford protein assay, suspending the sample in 25 µL of 4x Laemmli loading dye, bringing the volume to 100 µL using 1x radioimmunoprecipitation assay (RIPA) buffer, and boiling for 5 min at 95^°^C. 15 mg of protein were loaded into Invitrogen NuPAGE Bis-Tris Mini Protein Gels (cat# NP0335BOX) and run for 45 min at 165*V* using an Invitrogen Mini Gel Tank (cat# A25977) (Invitrogen, Waltham, MA) and BioRad PowerPac Basic (cat# 1645050) (BioRad, Hercules, CA). Transfer of the gel to nitrocellulose was performed using the Trans-Blot Turbo Nitrocellulose Transfer Pack (cat# 1704158) and the BioRad Transfer System (cat# 1704150) (BioRad, Hercules, CA). Primary antibodies for voltage-dependent anion channels (VDAC) (1:1000, cat# ab14734, Abcam, Waltham, MA) and enolase (1:1000, cat# sc-51880, Santa Cruz Technology, Dallas, TX) were used to determine the purity of the mitochondrial fractions. Mouse secondary antibody (1:10000, cat# 115-035-003, Jackson Immuno Research Laboratories, West Grove, PA) was diluted 1:10,000 in 5% milk in Tris-Buffered Saline with Tween (0.1% final concentration), and a Peirce ECL western kit (cat# 32132, Thermo, Waltham, MA) was used to visualize bands.

### Mitochondrial isolation and respirometry

Mitochondrial respiration was measured using a Clark electrode (Hansatech, Oxytherm + R with S1 Oxygen Electrode Disc, Norfolk, UK). Mitochondria were isolated using mitochondrial isolation buffer (MIB) (300mM ultrapure sucrose, [cat# S9378, Sigma, St. Louis, MO], 10 mM HEPES [cat #H-400, Goldbio, St. Louis, MO], and 2 mM EGTA [cat #E-217, Goldbio] in distilled water at pH 7.4) and oxygraph buffer (40 mM HEPES, 10 mM K_3_PO_4_ [cat #AC387685000, Fisher Scientific, Waltham, MA], 0.4 mM EDTA [cat #BP118, Fisher Scientific], 20 mM KCl [cat # 793590-500G, Sigma, St. Louis, MO], 5 mM anhydrous MgCl_2_ [cat #M8266, Sigma], 0.2% w/v BSA [cat #A3803, Sigma], and 500 mM sucrose [cat# S9378, Sigma]. Both MIB and oxygraph buffers were sterile filtered and stored at 4^°^C for up to a week (store at -20^°^C if stored longer).

Hearts from mice were perfused, excised and rinsed with ice cold MIB, and half used for mitochondrial isolation. All procedures were kept ice cold. Hearts were minced and homogenized using an overhead homogenizer (Caframo Universal, Model BDC3030, Georgian Bluffs, ON, CA) and tissue grinder with 8-inch pestle at 20 rpm (DWK, part #358029, Rockwood TN). The sample was further processed by centrifuging at 600 *g* for 2 min at 4^°^C. The supernatant (mitochondrial and cytosolic fraction) was centrifuged at 10,000 *g* for 5 min at 4^°^C with the pellet containing mitochondria and the supernatant containing the cytosolic fraction. We gently resuspended the pellet in 1 mL of MIB and centrifuged at 10,000 *g* for 5 min at 4^°^C a second time. The pellet was resuspended in 200 µL of oxygraph buffer for use Oxytherm analysis. Protein content of isolated mitochondria was determined by NanoDrop (ND2000, Thermo Scientific, Waltham, MA) and 250 µg of sample was added to the Oxytherm chamber. We first acquired baseline respiration (State 1) in the absence of any substrate. For State 2, in quick succession we added 2 µL 2 M L-glutamic acid monosodium hydrate (SKU: 02194677, MP Biomedicals, Santa Ana, CA), 2.5 µL 400 mM L-malic acid (cat# J63221, Thermo Fisher, Waltham, MA), 2 µL 1 M succinic acid (cat# 97061-122, VWR Life Sciences, Radnor, PA) diluted in water with a pH 7.4. For State 3 we added 4 µL 250 mM ADP (cat# 97061-106, VWR Life Sciences) diluted in Oxytherm buffer at a pH 7.4. For State 4, we added 5 µL 10 µM oligomycin (cat# O4876, Millipore Sigma, St. Louis, MO) dissolved in 100% absolute ethanol (cat# AAP-111000200CSGL, Capitol Scientific, Austin, TX). And for Background we added 2µL of 5 mM antimycin A (cat# A8674, Sigma) dissolved in 100% ethanol.

For analysis, we calculated the slope between two timepoints 60 s apart when the chamber was most stable. For State 3, we chose a timepoint immediately prior to adding ADP with the second timepoint being 60 s later to calculate the slope. That gives a slope of saturating conditions of ADP. For State 2, 3 and 4 we accounted for Background respiration by adding the respiration in the presence of Antimycin A. For the net oxidative phosphorylation capacity, we subtracted State 4 from State 3. For the respiratory control ratio (RCR), we divided State 3 (numerator) by State 4 (denominator).

### Chromatin immunoprecipitation sequencing (ChIPseq)

Hearts representing mean myocarditis inflammation scores for each group were chosen for ChIPseq and sent to the Mayo Clinic Epigenomics and Sequencing Core. After collection of hearts from mice at harvest, ½ was fixed in 10% phosphate-buffered saline (as above) and the other half was flash frozen in liquid nitrogen and stored at -80^°^C for ChIPseq. Samples were thawed and their wet weight was determined to determine antibody titer (3.5 µL antibody per 50 mg tissue). Thawed tissue was immediately fixed using a formaldehyde-based buffer fixative, homogenized (i.e., Polytron based homogenization), incubated in fixative at room temperature for 10 min, and centrifuged to pellet nuclei. Fixed nuclei were sonicated at 4^°^C to achieve major chromatin product length of 100–200 base pairs. ChIPs were processed using magnetic bead conjugation (cat# 10001D, ThermoFisher). After pull-down, chromatin was eluted and de-crosslinked prior to ethanol precipitation purification of DNA. The ChIP antibody (cat# PA5-28390, ThermoFisher) was tested for specific enrichment of ERRα known binding regions for ESRRA and PDK4 using additional frozen heart tissue from the same harvests that would not be used for ChIPseq. This was accomplished with the following primers and mCherry probes with fluorescence quantitative PCR, correct product length was also evaluated:

ESRRA-Fwd1: GGACCCTCAAGTGGAGAAGC.

ESRRA-Rev1: CGTGTCTCACCTCTGCCTTT.

ESRRA primer set 1 product length: 81 base pairs.

ESRRA-Fwd2: TAGCCCTGGTTTGCGAGTTC.

ESRRA-Rev2: ACCGCAGTGACCTTGAGTTT.

ESRRA primer set 2 product length: 71 base pairs.

PDK4-Fwd1: AATGTCACGCATTCCTAGCCA.

PDK4-Rev1: GGCTACTGTAAAAGTCCCGCT.

PDK4 primer set 1 product length: 93 base pairs.

PDK4-Fwd2: GGATAGATCCCAGGTCGCTA.

PDK4-Rev2: TTTCTGGCTAGGAATGCGTGA.

PDK4 primer set 2 product length: 97 base pairs.

After ideal chromatin product size and antibody/ChIP enrichment over input for ERRα positive regions were confirmed, input samples and ChIPs libraries were prepared sent to the Mayo Clinic Sequencing core for paired-end sequencing using a NovaSeq 6000 SP.

### ChIPseq analysis

After sequencing of ERRα ChIPs and inputs, quality control was assessed using fastqc and ENCODE quality metrics. BWA was used for mapping and peaks were called with MACS2C. Differential binding analysis of comparator groups was accomplished with DiffBind. Concatenation of ERRα binding predictions was performed by manually searching for and selecting nuclear encoded mitochondrial electron transport chain gene names comparing males with myocarditis and females with myocarditis, selecting only significantly significant results (*p* < 0.05) and manually comparing to RNAseq and TRANSFAC data.

### Statistical analysis

Normally distributed data comparing two groups, determined with Prism, were analyzed using a 2-tailed Student’s *t* test. Multiple comparison analysis was performed by ANOVA with each group compared to the corresponding control group; 2-way ANOVA was used to determine the effect of sex vs. disease (myocarditis) using a main effects model. Multiple comparisons were performed with Holm-Sidak. Outlier analysis/exclusion was performed with ROUT (Q = 2%). Data are displayed as mean ± SEM. A value of *p* < 0.05 was considered significant. Adjusted p-values (from Prism) were used for multiple comparisons.

## Electronic supplementary material

Below is the link to the electronic supplementary material.

Supplementary Material 1: Additional File 1: Figure S1. Other pathways/genesets from F-MYO vs M-MYO Enrichment Mapping. Figure S2. Row normalized heatmaps from GSEA analysis comparing mitochondrial pathways for F-MYO vs M-MYO. Figure S3. Enrichment mapping results from Metascape for F-CON comparing F-CON vs F-MYO. Figure S4. Enrichment mapping results from Metascape for F-MYO comparing F-CON vs F-MYO. NMD = nonsense mediated decay, EJC = exon junction complex. Figure S5. Enrichment mapping results from Metascape for M-CON comparing M-CON vs M-MYO. Figure S6. Enrichment mapping results from Metascape for M-MYO comparing M-CON vs M-MYO. Figure S7. Verification of mitochondrial fraction purity. Figure S8. Background respiration in the presence of antimycin A measured by Clark electrode. Figure S9. Females with myocarditis express higher levels of mitochondrial master regulators PGC1α and NRF1. Table S1. gProfiler enrichment results comparing males and females with myocarditis Table S2. Published sex differences in cardiac mitochondrial properties

Supplementary Material 2: Additional File 2: Mapped ChIPseq raw counts and statistics for Figure 13. iF = F-MYO; iM = M-MYO

Supplementary Material 3: Additional File 3: RNAseq differential expression comparing F-CON and M-CON. uBAM = M-CON; uBAF = F-CON

Supplementary Material 4: Additional File 4: RNAseq differential expression comparing F-CON and F-MYO. uBAF = F-CON; iBAF = F-MYO

Supplementary Material 5: Additional File 5: RNAseq differential expression comparing M-CON and M-MY. uBAM = M-CON; iBAM = M-MYO

Supplementary Material 6: Additional File 6: RNAseq differential expression comparing F-MYO and M-MYO. iBAM = M-MYO; iBAF = F-MY0

## Data Availability

Data used for RNA sequencing and chromatin immunoprecipitation sequencing can be found in the supplemental/additional files. Other requests for data used in this manuscript can be addressed to the corresponding author and are available upon reasonable request.
