# Supplementary material for: Sex differences in mitochondrial gene expression during viral myocarditis
Source: Biol Sex Differ. 2024 Dec 18;15:104. doi: 10.1186/s13293-024-00678-0 (PMC11657264; doi:10.1186/s13293-024-00678-0)
Supplement: Supplementary file 1 — Supplementary Material 1: Additional File 1: Figure S1. Other pathways/genesets from F-MYO vs M-MYO Enrichment Mapping. Figure S2. Row normalized heatmaps from GSEA analysis comparing mitochondrial pathways for F-MYO vs M-MYO. Figure S3. Enrichment mapping results from Metascape for F-CON comparing F-CON vs F-MYO. Figure S4. Enrichment mapping results from Metascape for F-MYO comparing F-CON vs F-MYO. NMD = nonsense mediated decay, EJC = exon junction complex. Figure S5. Enrichment mapping results from Metascape for M-CON comparing M-CON vs M-MYO. Figure S6. Enrichment mapping results from Metascape for M-MYO comparing M-CON vs M-MYO. Figure S7. Verification of mitochondrial fraction purity. Figure S8. Background respiration in the presence of antimycin A measured by Clark electrode. Figure S9. Females with myocarditis express higher levels of mitochondrial master regulators PGC1α and NRF1. Table S1. gProfiler enrichment results comparing males and females with myocarditis Table S2. Published sex differences in cardiac mitochondrial properties [file 13293_2024_678_MOESM1_ESM.docx]

**Figure S1.** Other pathways/gene sets from F-MYO vs. M-MYO Enrichment Mapping


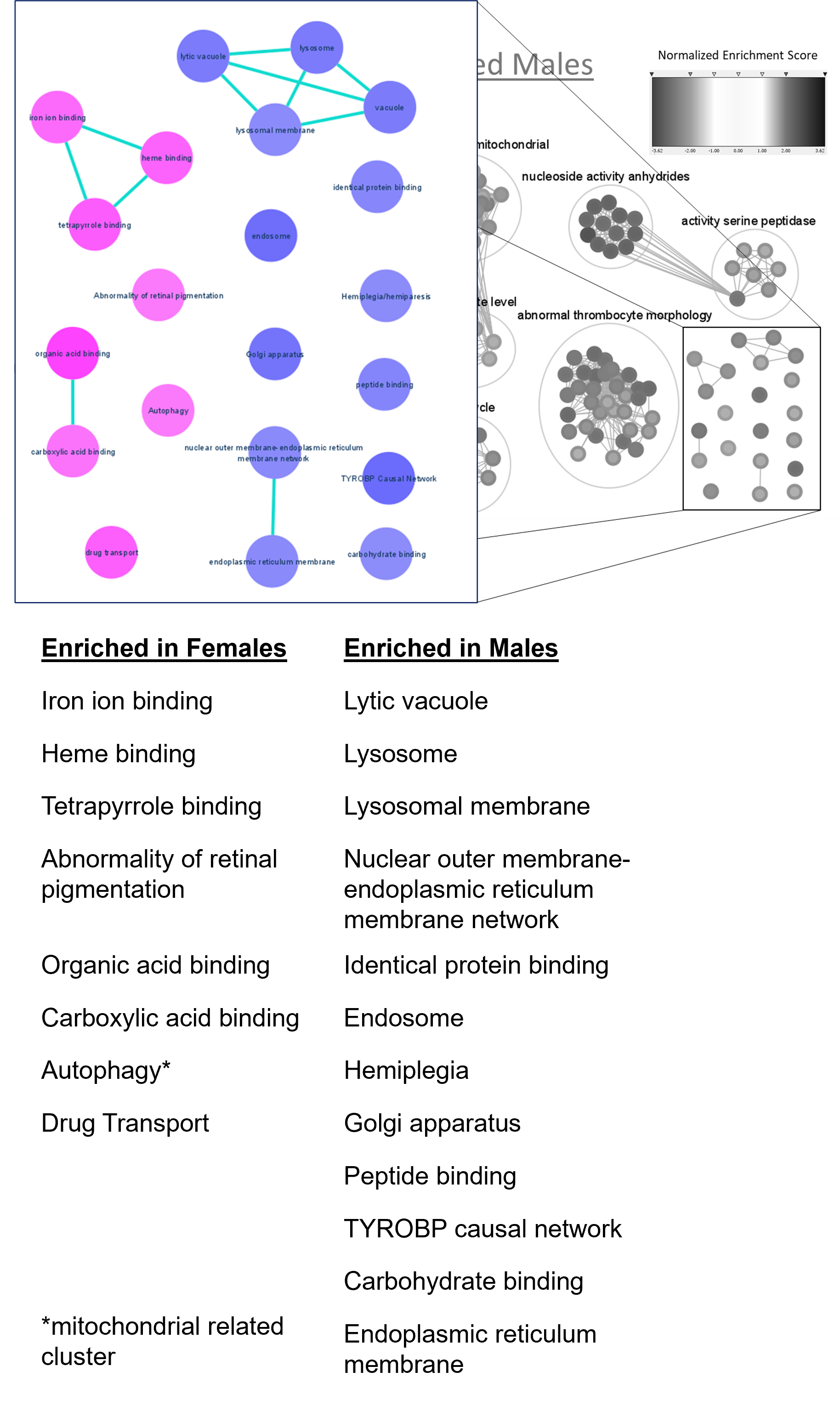


**Figure S2.** Row normalized heatmaps from GSEA analysis comparing mitochondrial pathways for F-MYO vs M-MYO


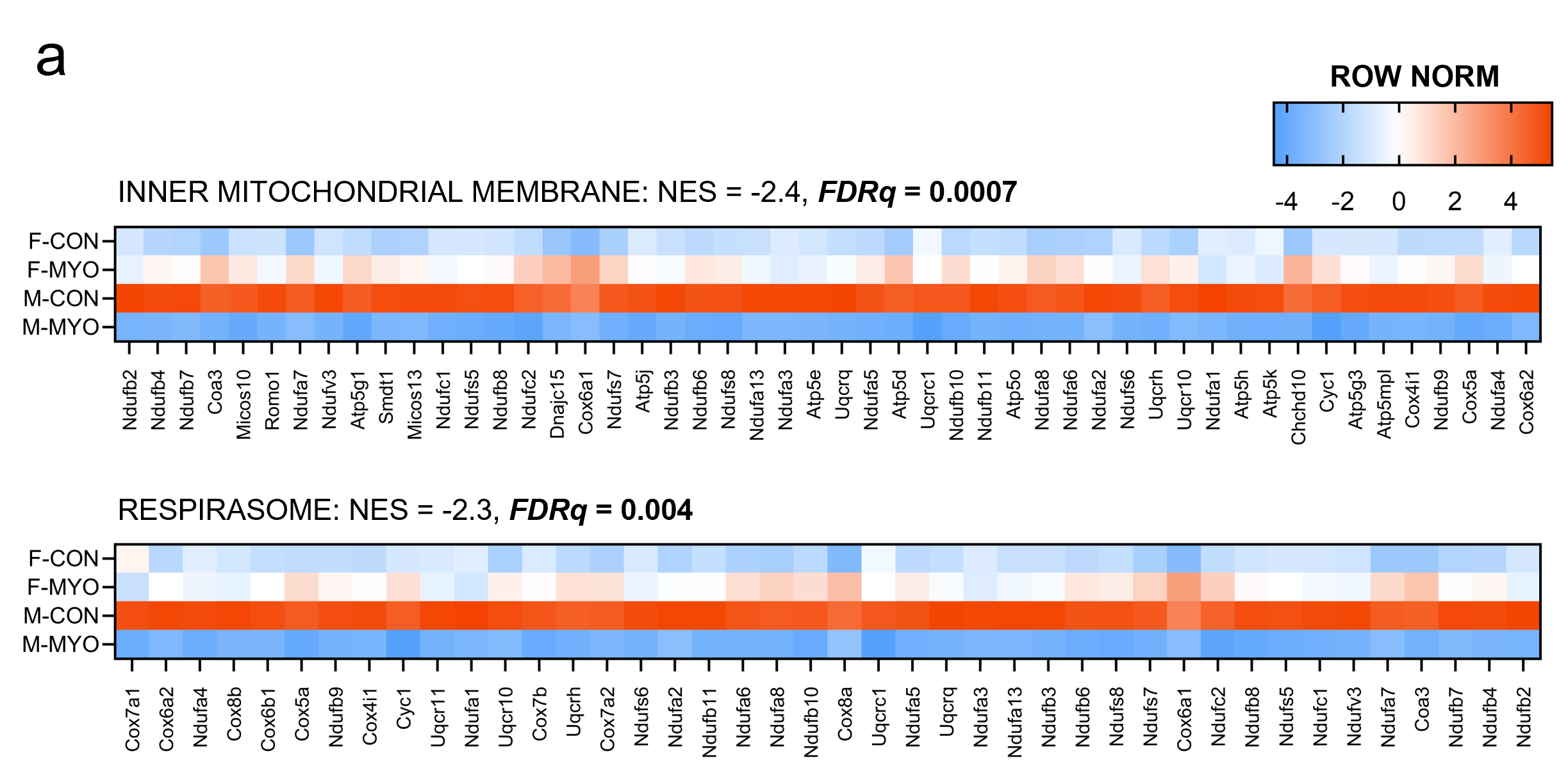


**Figure S3.** Enrichment mapping results from Metascape for F-CON comparing F-CON vs F-MYO

**
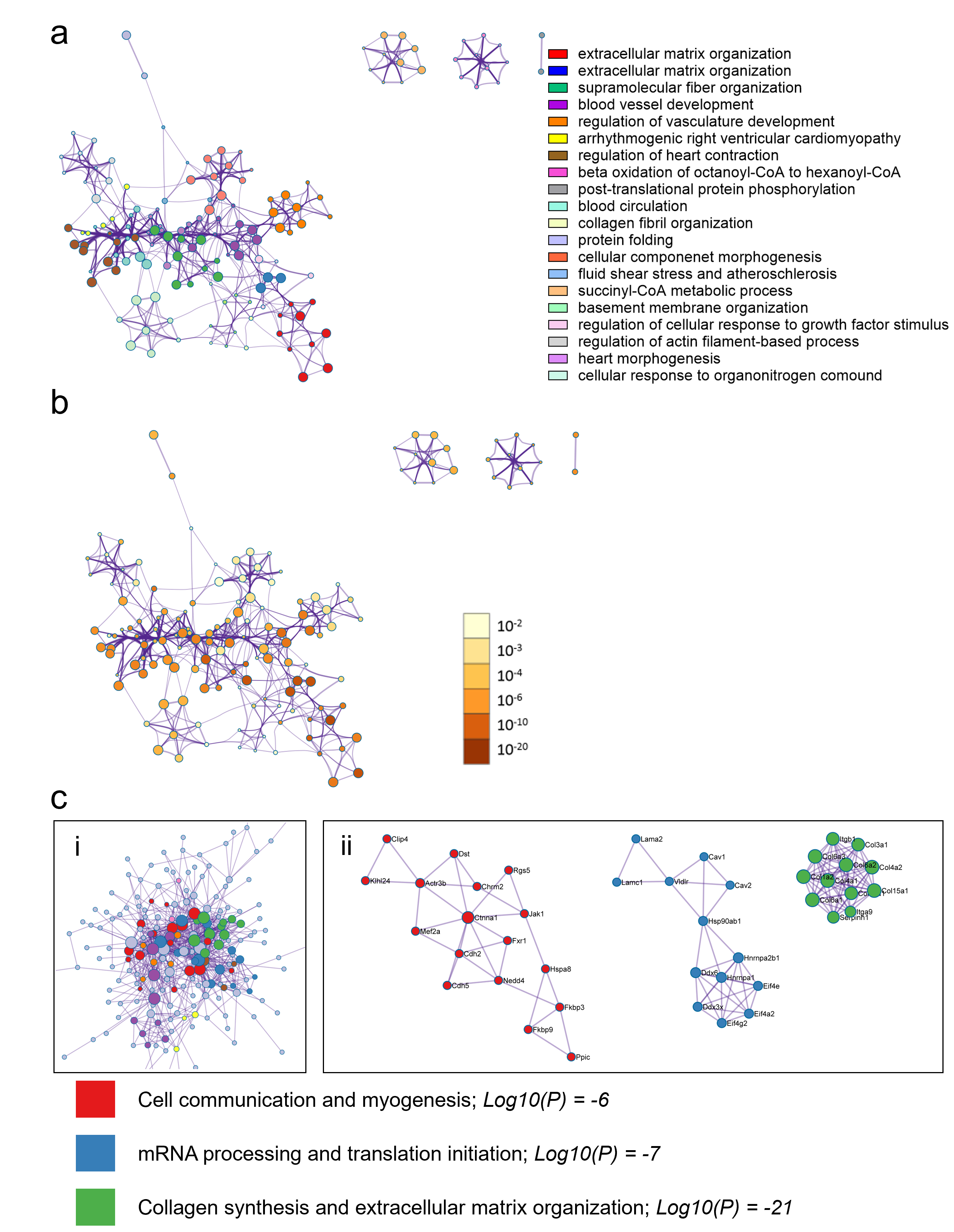
**

**Figure S4.** Enrichment mapping results from Metascape for F-MYO comparing F-CON vs F-MYO. NMD = nonsense mediated decay, EJC = exon junction complex


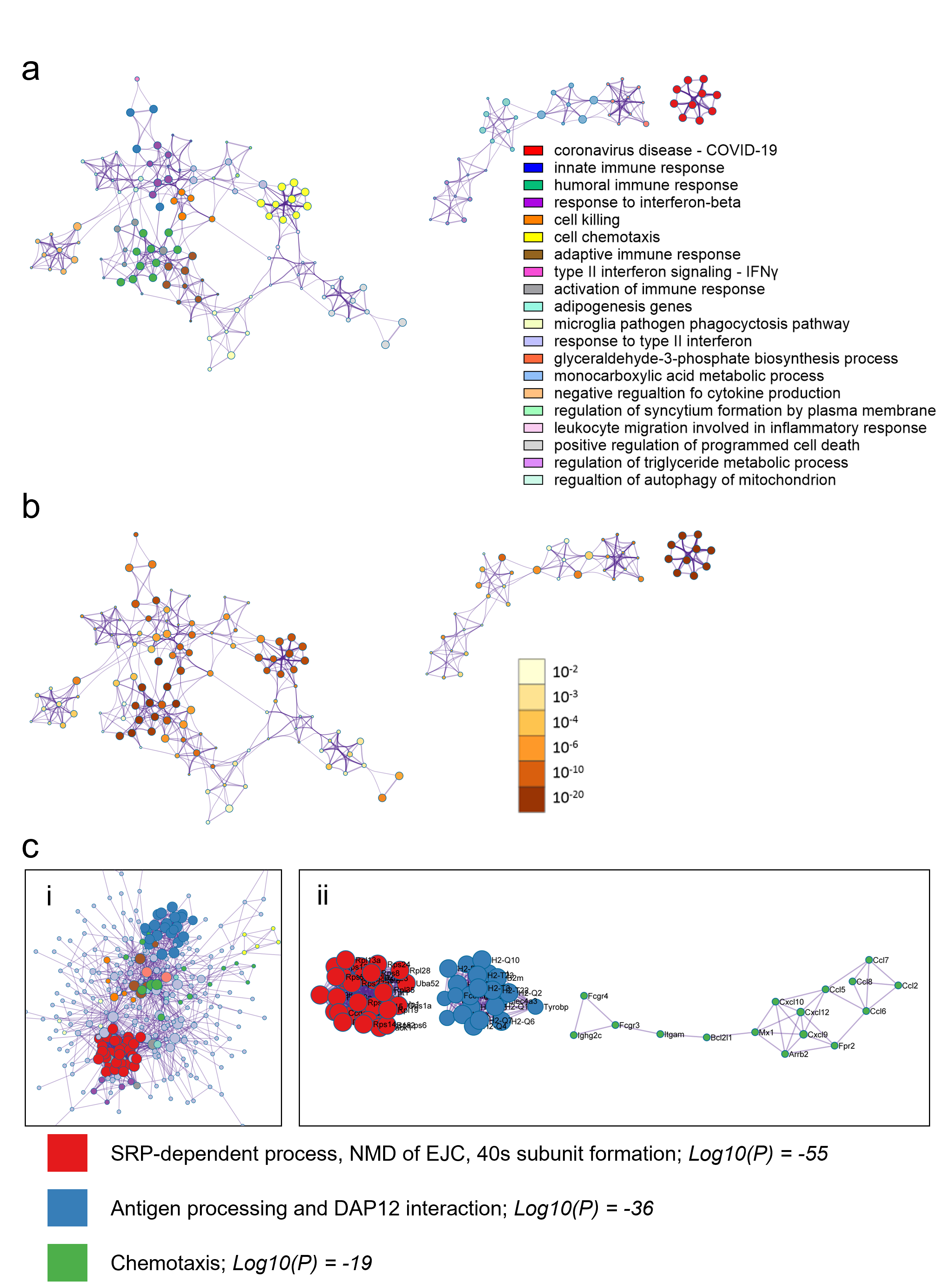


**Figure S5.** Enrichment mapping results from Metascape for M-CON comparing M-CON vs M-MYO


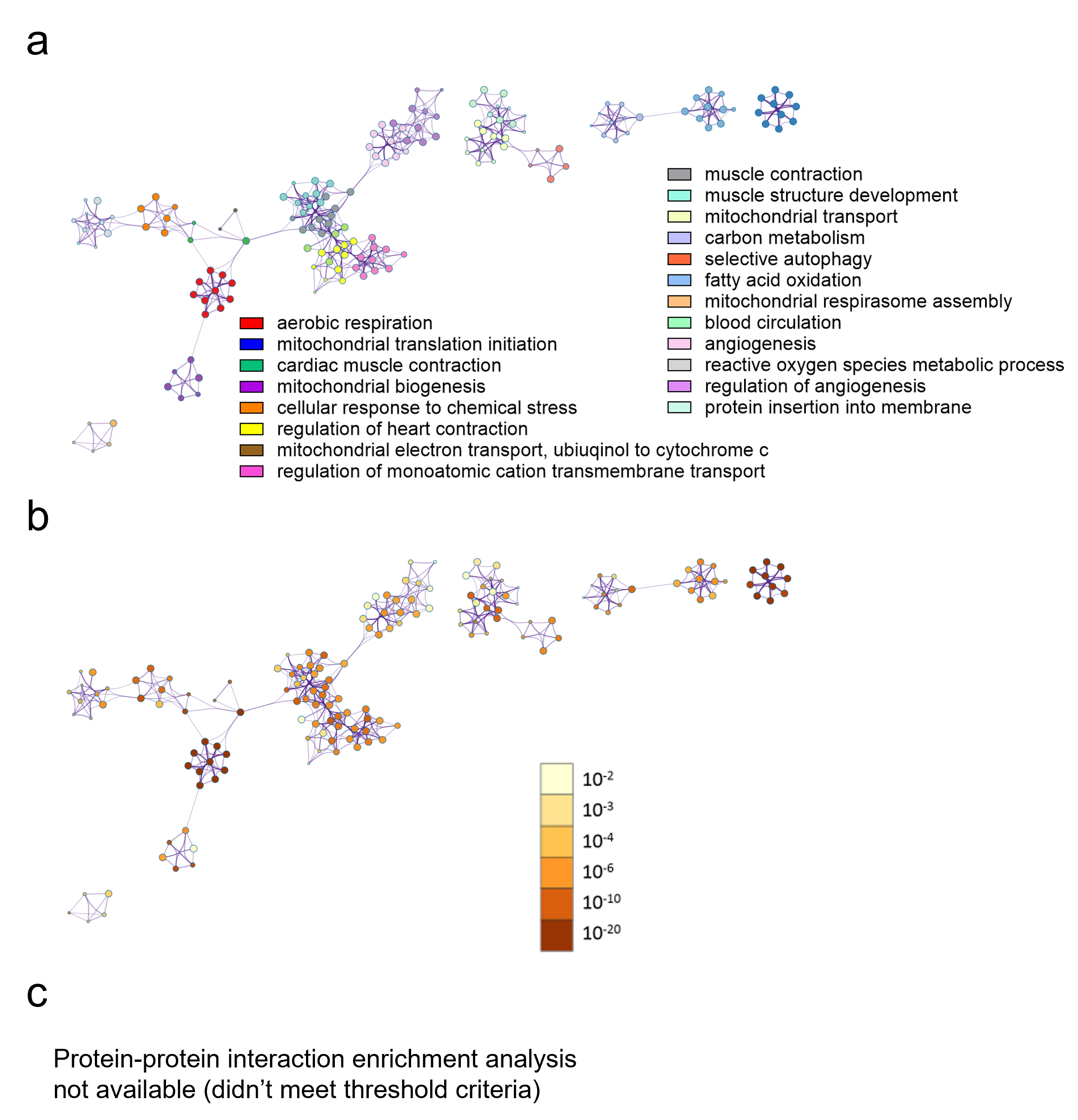


**Figure S6.** Enrichment mapping results from Metascape for M-MYO comparing M-CON vs M-MYO

**
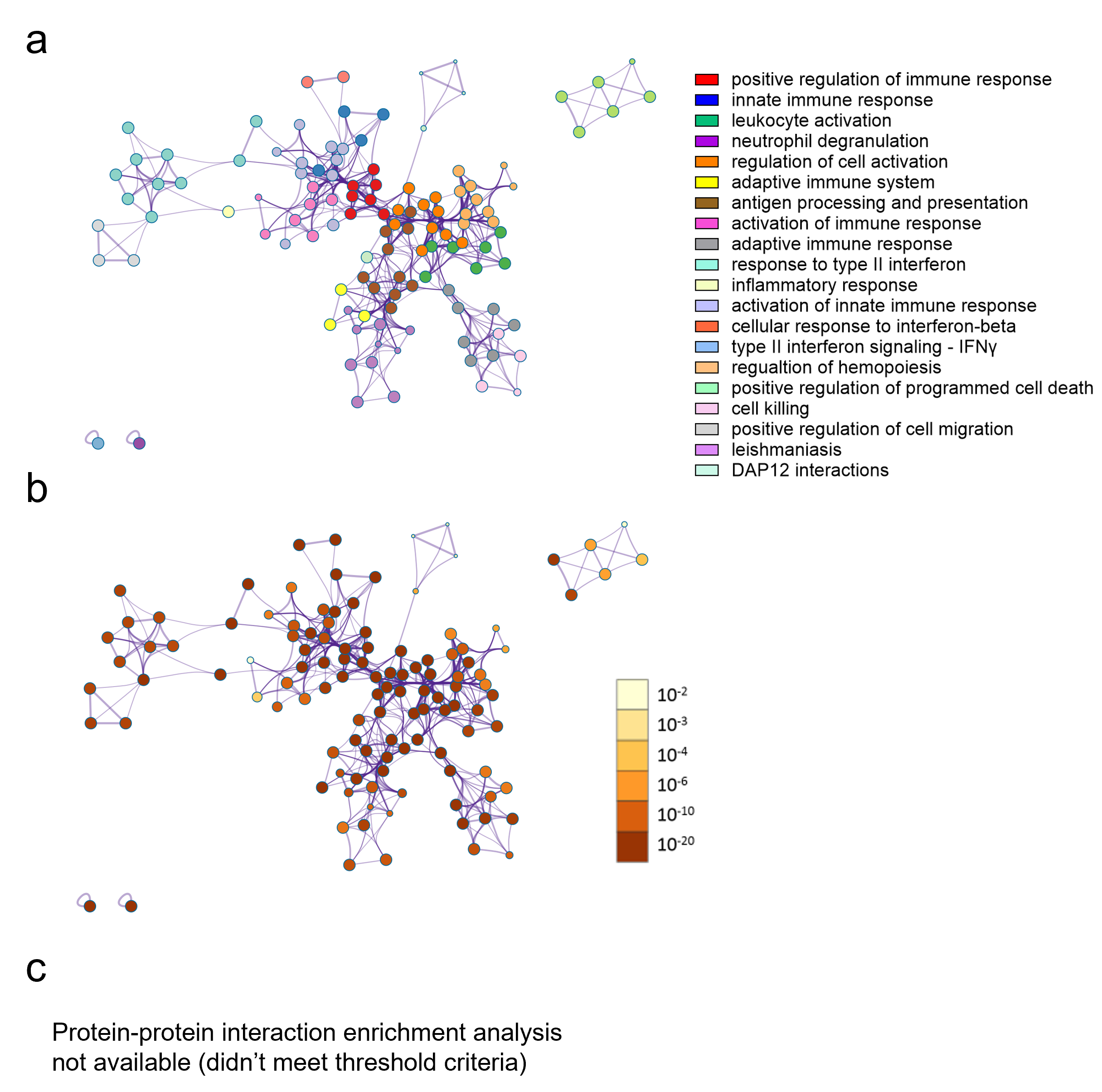
**

a **Blot 1**

1 2 3 4 5 6 7 8 9

52kDa

38kDa


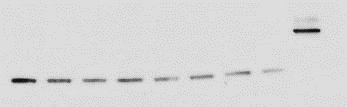


Enolase

MBP-1

b **Blot 2**


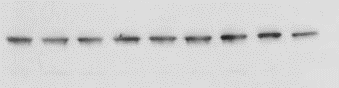


38kDa

31kDa

VDAC

**Figure S7.** Verification of mitochondrial fraction purity

Mitochondria were isolated from cardiac myocytes at day 10 during myocarditis using differential centrifugation, and 15μg protein loaded per well. **a**, Western blot showing enolase, a 48kDa protein, binding in the cytosolic fraction but not the mitochondrial fraction. Banding observed at ~36kDa we suspect is the alternatively spliced product of enolase, c-Myc promoter-binding protein 1 (MPB-1), which localized to the mitochondrial fractions (Czogalla, 2021; Cancemi, 2019; Feo, 2000). **b**, Western blot showing voltage dependent anion channel (VDAC) binding at ~33kDa, which is present in mitochondria, verifying successful isolation of cardiac mitochondria.

1: Pooled mitochondrial fraction from heart during myocarditis at day 10.

2: Pooled mitochondrial fraction from the heart of uninfected control mice.

3: Mitochondrial fraction from the heart of a representative male with myocarditis.

4: Mitochondrial fraction from the heart of a representative female with myocarditis.

5: Mitochondrial fraction from the heart of a representative male with myocarditis.

6: Mitochondrial fraction from the heart of a representative female with myocarditis.

7: Mitochondrial fraction from the heart of a representative male with myocarditis.

8: Mitochondrial fraction from the heart of a representative female with myocarditis.

9: Pooled cytosolic fraction from heart during myocarditis at day 10.

Czogalla B, Partenheimer A, Badmann S, Schmoeckel E, Mayr D, Kolben T, Beyer S, Hester A, Burges A, Mahner S, Jeschke U, Trillsch F. Nuclear Enolase-1/ MBP-1 expression and its association with the Wnt signaling in epithelial ovarian cancer. Transl Oncol. 2021 Jan;14(1):100910. doi: 10.1016/j.tranon.2020.100910. Epub 2020 Oct 14. PMID: 33069100; PMCID: PMC7569221.

Cancemi P, Buttacavoli M, Roz E, Feo S. Expression of Alpha-Enolase (ENO1), Myc Promoter-Binding Protein-1 (MBP-1) and Matrix Metalloproteinases (MMP-2 and MMP-9) Reflect the Nature and Aggressiveness of Breast Tumors. Int J Mol Sci. 2019 Aug 14;20(16):3952. doi: 10.3390/ijms20163952. PMID: 31416219; PMCID: PMC6720302.

Feo S, Arcuri D, Piddini E, Passantino R, Giallongo A. ENO1 gene product binds to the c-myc promoter and acts as a transcriptional repressor: relationship with Myc promoter-binding protein 1 (MBP-1). FEBS Lett. 2000 May 4;473(1):47-52. doi: 10.1016/s0014-5793(00)01494-0. PMID: 10802057.

**Figure S8.** Background respiration in the presence of antimycin A measured by Clark electrode

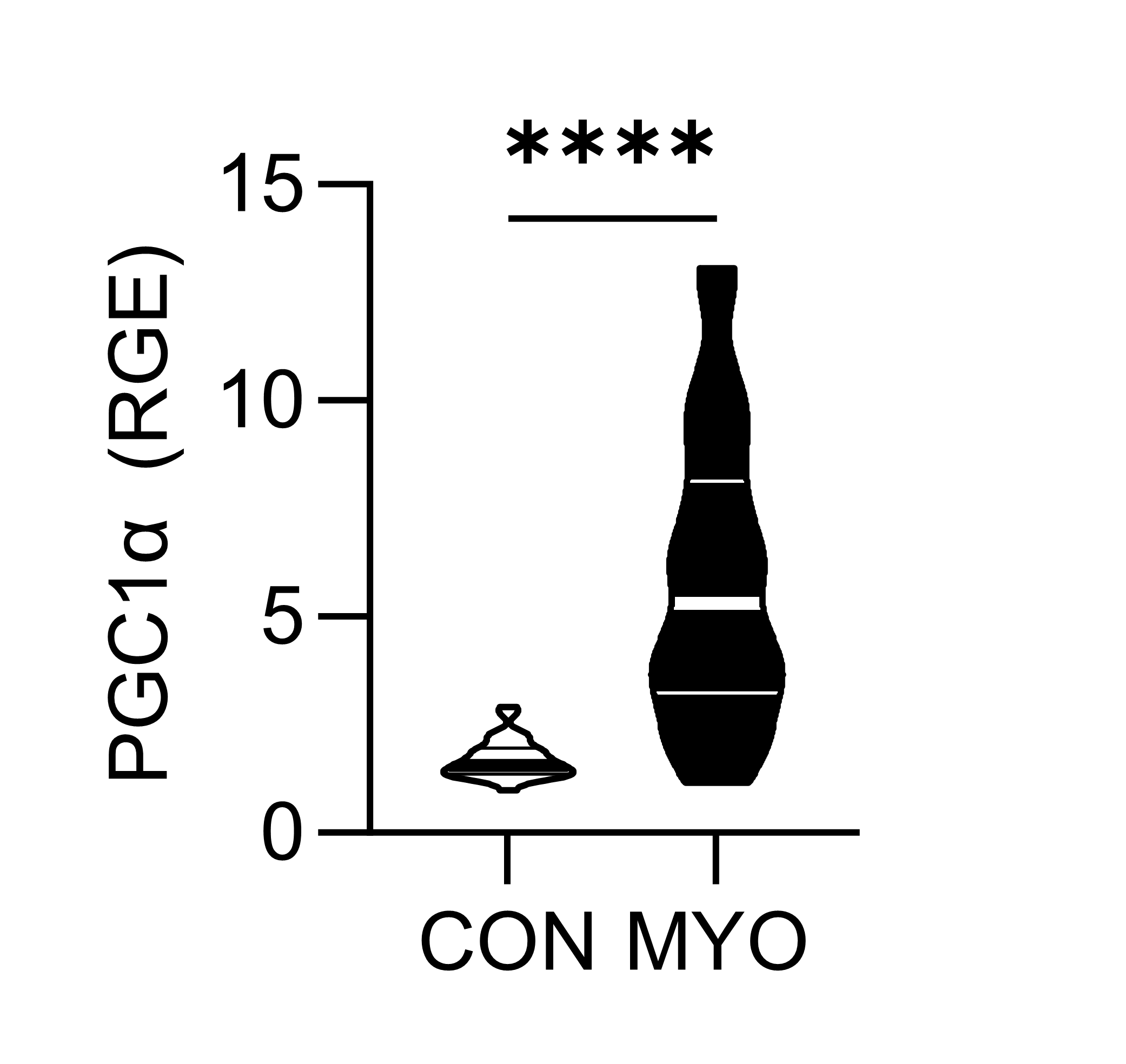

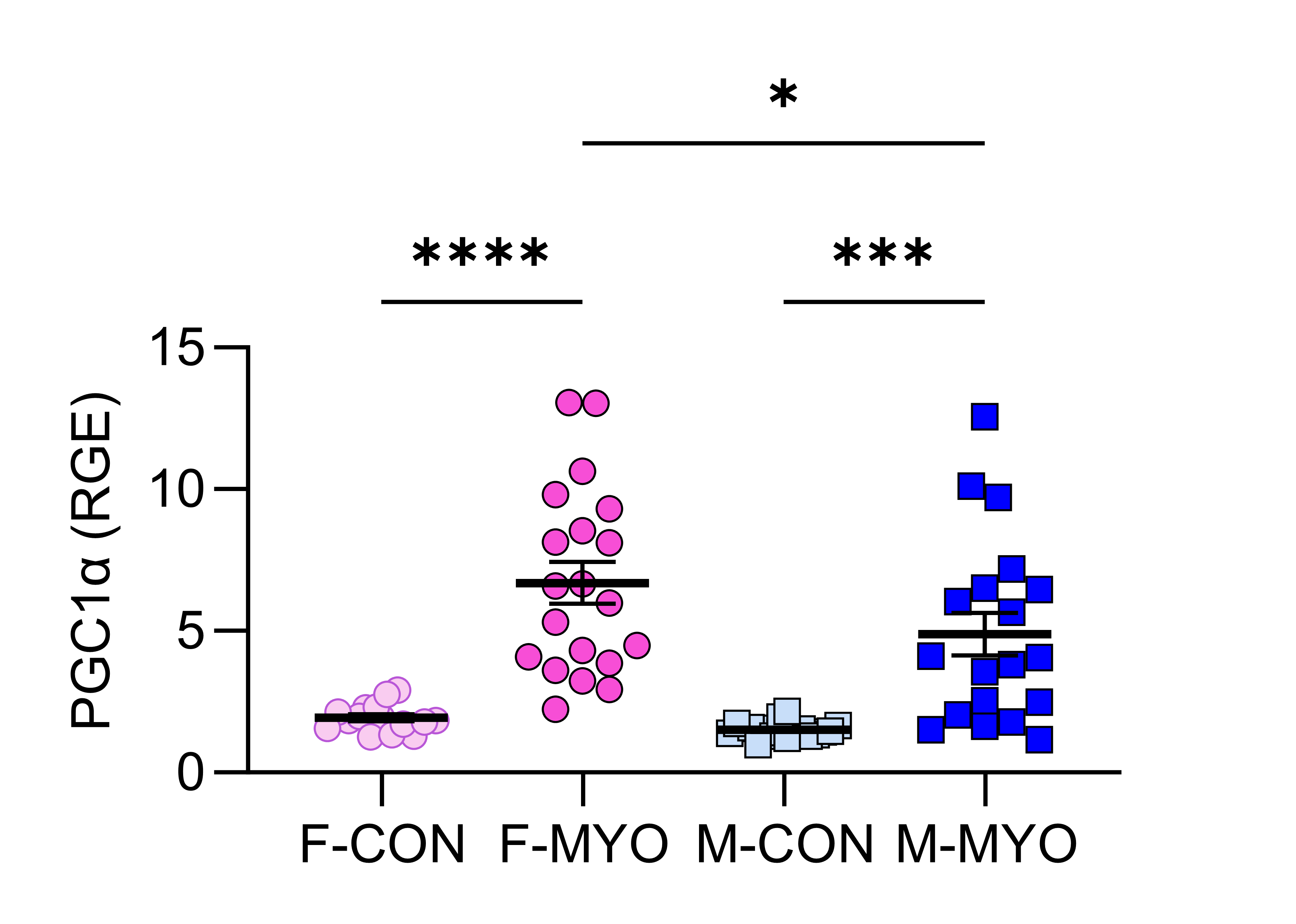

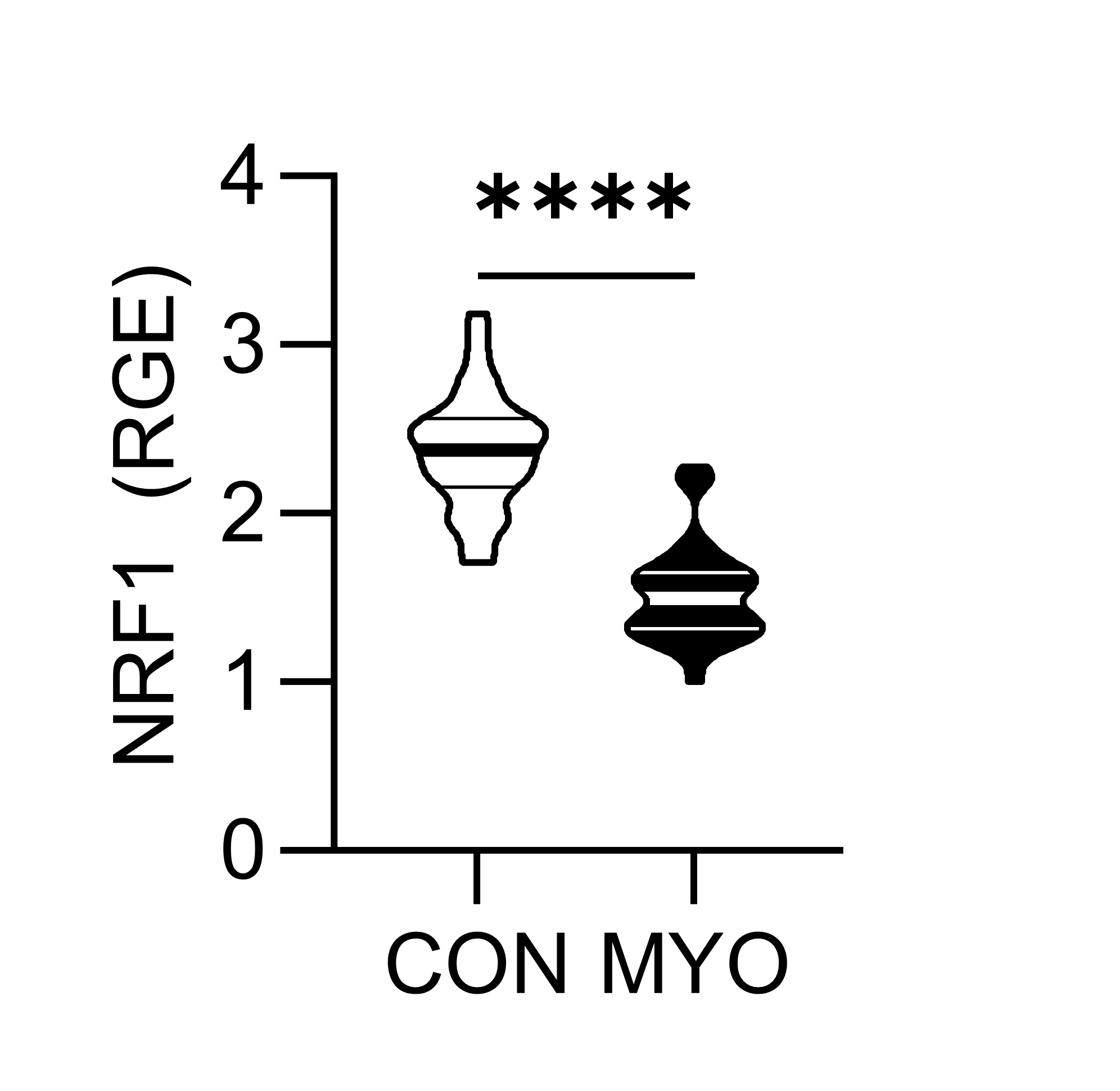

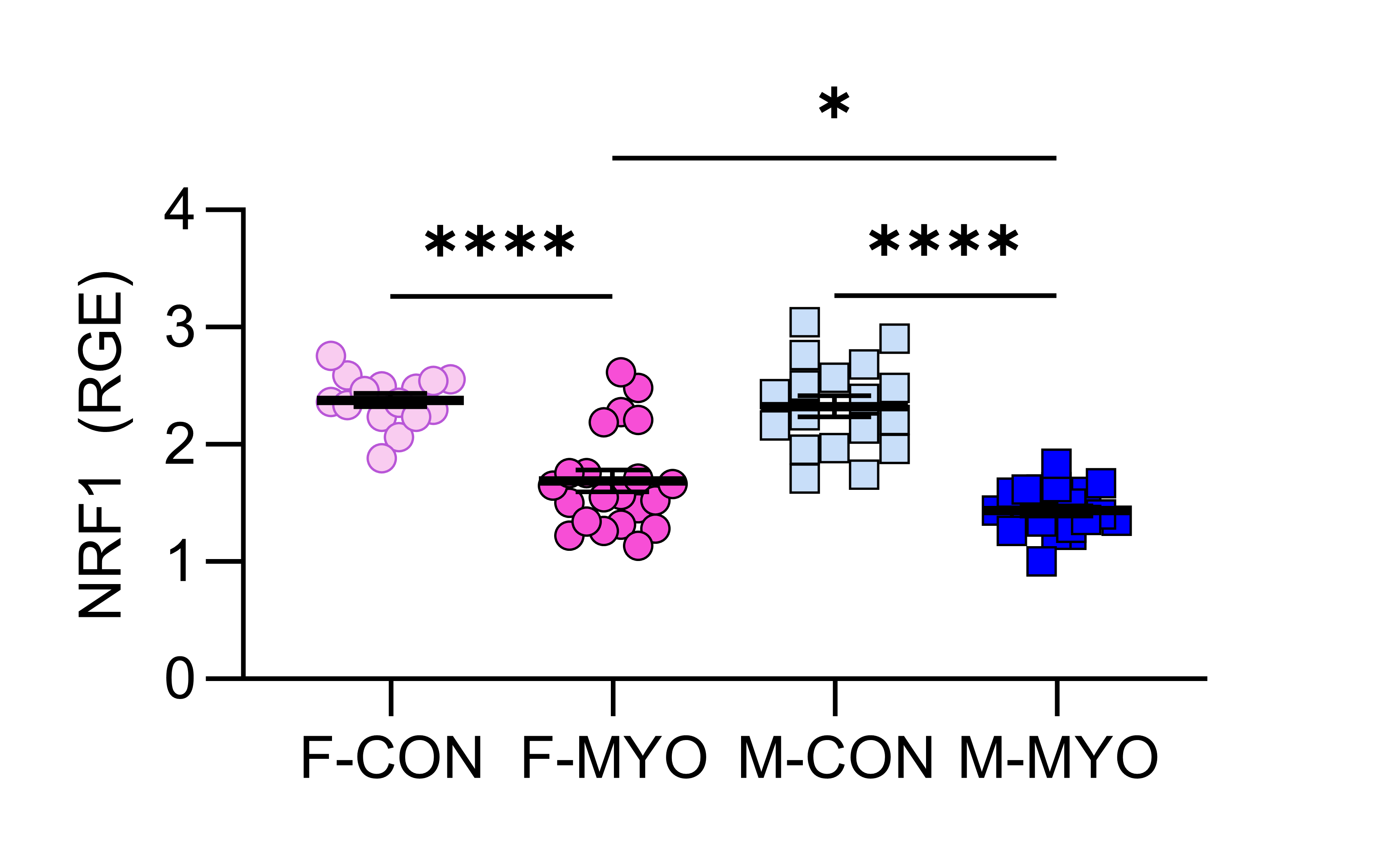


a

c

b

d

**Figure S9.** **Females with myocarditis express higher levels of mitochondrial master regulators PGC1α and NRF1 than males.** Relative gene expression (RGE) for F-CON (*n =* 15), F-MYO (*n =* 20-21), M-CON (*n =* 17-18), and M-MYO (*n =* 19-20) for **a-b,** PGC1α; **c,d,** NRF1. **b,d,** 2-way ANOVA was used to assess significance between groups, **p <* 0.05, **** *p* < 0.0001.

**Table S1.** gProfiler enrichment results comparing males and females with myocarditis

| **Source** | **Term Name** | **Term ID** | **P Adj***^a^* |
| --- | --- | --- | --- |
| GO:MF | electron transfer activity | GO:0009055 | 2.68 E-37 |
| GO:MF | oxidoreductase activity | GO:0016491 | 5.30 E-17 |
| GO:MF | NADH dehydrogenase (ubiquinone) activity | GO:0008137 | 7.21 E-17 |
| GO:MF | NADH dehydrogenase activity | GO:0003954 | 7.42 E-17 |
| GO:MF | NADH dehydrogenase (quinone) activity | GO:0050136 | 1.95 E-16 |
| GO:BP | electron transport chain | GO:0022900 | 1.62 E-44 |
| GO:BP | aerobic respiration | GO:0009060 | 1.55 E-42 |
| GO:BP | cellular respiration | GO:0045333 | 1.19 E-41 |
| GO:BP | oxidative phosphorylation | GO:0006119 | 5.86 E-40 |
| GO:BP | generation of precursor metabolites and energy | GO:0006091 | 1.23 E-39 |
| GO:CC | mitochondrial inner membrane | GO:0005743 | 5.25 E-62 |
| GO:CC | inner mitochondrial membrane protein complex | GO:0098800 | 6.66 E-60 |
| GO:CC | respirasome | GO:0070469 | 6.78 E-58 |
| GO:CC | mitochondrial protein-containing complex | GO:0098798 | 1.43 E-57 |
| GO:CC | organelle inner membrane | GO:0019866 | 1.31 E-55 |
| KEGG | Oxidative phosphorylation | KEGG:00190 | 6.37 E-37 |
| KEGG | Diabetic cardiomyopathy | KEGG:05415 | 7.62 E-30 |
| KEGG | Thermogenesis | KEGG:04714 | 1.51 E-24 |
| KEGG | Prion disease | KEGG:05020 | 3.08 E-23 |
| KEGG | Non-alcoholic fatty liver disease | KEGG:04932 | 1.27 E-22 |
| REACT | The citric acid (TCA) cycle and respiratory electron transport | R-MMU-1428517 | 1.66 E-35 |
| REACT | Respiratory electron transport, ATP synthesis*^b^* | R-MMU-163200 | 3.77 E-35 |
| REACT | Respiratory electron transport | R-MMU-611105 | 2.34 E-30 |
| REACT | Complex I biogenesis | R-MMU-6799198 | 1.66 E-20 |
| REACT | Formation of ATP by chemiosmotic coupling | R-MMU-163210 | 2.16 E-05 |

*^a^* Abbreviations: Adj, adjusted; GO:MF, gene ontology molecular function; GO:BP, gene ontology biological process; GO:CC, gene ontology cellular compartment; KEGG, Kyoto encyclopedia of genes and genomes; REACT, reactome.

*^b^* Full name: Respiratory electron transport, ATP synthesis by chemiosmotic coupling, and heat production by uncoupling proteins.

**Table S2.** Published sex differences in cardiac mitochondrial properties*^a^*

| **Mitochondrial Property** | | **Sex Difference** |
| --- | --- | --- |
| Mitochondrial content | F < M*^b^* | |
| Mitochondrial efficiency and differentiation | F > M | |
| Oxygen consumption rate (basal) | F = M | |
| Cardiolipin Content | F = M | |
| Glutamate/malate-stimulated respiration | F > M | |
| Other substrates-stimulated respiration | F = M | |
| ADP/O ratio | F > M | |
| Fatty acid utilization during exercise | F > M | |
| ROS production | F < M | |
| Calcium uptake rate | F < M | |
| Calcium retention rate | F > M | |

*^a^* Modified from Ventura-Clapier, R., Moulin, M., Piquereau, J., Lemaire, C., Mericskay, M., Veksler, V., and Garnier, A. (2017). Mitochondria: a central target for sex differences in pathologies. Clin Sci (Lond) *131*, 803-822. 10.1042/CS20160485.

*^b^* Abbreviations: ADP, adenosine diphosphate ; F, female; M, male; ROS, reactive oxygen species.
